# RNA-Seq exploration of the influence of stress on meat quality in Spanish goats

**DOI:** 10.1038/s41598-022-23269-8

**Published:** 2022-11-29

**Authors:** Aditya Naldurtiker, Phaneendra Batchu, Brou Kouakou, Thomas H. Terrill, Arshad Shaik, Govind Kannan

**Affiliations:** Agricultural Research Station, Fort Valley State University, 1005 State University Drive, Fort Valley, GA 31030 USA

**Keywords:** Biochemistry, Biotechnology, Cell biology, Molecular biology, Physiology

## Abstract

Studies exploring the transcriptome of stress and its effects on meat quality are very limited, particularly in goats. Fifty-four male Spanish goats (8-mo old; BW = 29.7 ± 2.03 kg) were randomly subjected to one of three treatments (TRT; n = 18 goats/treatment): (1) transported for 180 min, (2) transported for 30 min, or (3) held in pens (control) to analyze the transcriptome of stress and meat quality in goats using RNA-seq technology. Blood samples were collected before and after treatment, and meat samples were collected after humane slaughter for stress hormone, meat quality (*Longissimus dorsi*), and transcriptomic analysis. Plasma epinephrine concentrations were higher (*P* < 0.01) in 180 min and 30 min groups compared to the control group; however, norepinephrine concentrations were not affected by the treatment. Muscle glycogen concentrations (15 min postmortem) were lower (*P* < 0.01) in both 30 min and 180 min groups compared to the control group. Calpastatin levels were higher (*P* < 0.01) in 180 min and 30 min groups than the control group. Warner–Bratzler shear force values of loin chops were the highest in the 180 min group (4 ± 0.15, kg), lowest in the control group (3.51 ± 0.10, kg), and intermediate in the 30 min group (3.78 ± 0.09, kg; *P* < 0.01) both at day 1 and day 6 aging time. Additionally, desmin levels of day 6 samples were lowest in the control group, highest in 180 min group, and intermediate in 30 min group (*P* < 0.05). RNA-seq results showed that a total of 10,633 genes were differentially expressed (5194 up regulated; 5439 down regulated) among all comparisons (blood and day 1 and day 6 muscle samples). Among these differentially expressed genes (DEGs), KLF9, AMPK, FOXO3, PTX3, GADD45, PTPN1, CASP7, MAPK4, HSPA12A, and JAK-STAT were probably associated with the effects of stress on skeletal muscle proteins and involved in biological process such as cellular response to corticosteroid stimulus, endoplasmic reticulum stress, insulin resistance, DNA repair, apoptosis, MAPK cascade and regulation of proteolysis. The KEGG analysis revealed that AMPK and JAK-SAT signaling pathways and autophagy were among the top 20 enriched pathways in our treatment comparisons. The results provide an understanding of the genes and pathways involved in stress responses and related changes in postmortem muscle metabolism and meat quality characteristics in goats.

## Introduction

Exposure to stressors such as handling, movement of animals during loading and unloading, food deprivation, extreme changes in weather, and poor ventilation, prior to processing can cause severe metabolic changes that have shown to negatively affect meat quality characteristics in small ruminants^1^. Catecholamines that are released due to preslaughter stress deplete glycogen stores in the body, resulting in higher muscle pH postmortem and darker meat^2^. Stress related to transportation and isolation have been reported to reduce muscle glycogen levels in small ruminants, and the effect is more prominent in older than younger animals^3^.

Severe preslaughter stress in goats can result in tougher meat; however, aging of meat can improve tenderness due to proteolysis^3,4^. Skeletal muscle proteins, such as calpains (calpain 1 and calpain 2), caspases (caspase-3, caspase-7, caspase-8, and caspases-9), and calpastatin are responsible for postmortem tenderization of meat^5,6^. Calpains which are activated by calcium overload in the cytosol of the cell due to stress, activates caspases 12, which further activates caspases-9 and caspases-3, leading to cell apoptosis^7^. The calpain specific inhibitor calpastatin is responsible for lower degradation of myofibrillar proteins^8^. Desmin, an important cytoskeleton protein in muscle has been shown to be a substrate for the action of calpains^9^. Desmin concentration was found to be lower in the cortisol-administered pigs compared to the control animals^10^. Furthermore, low animal welfare practices have been reported to interfere with troponin-T degradation during aging in *Longissimus* muscle in suckling goats^11^.

RNA-sequencing is a robust technology for comparing gene expressions and discovering novel transcripts in animals. Transcriptome analysis of *Longissimus dorsi* and *Biceps femoris* cuts from pig carcasses have shown the molecular mechanisms that affect meat quality traits^12,13^. Transcriptomic profiling of meat from Liaoning cashmere goats and Zwuling black goats has helped researchers identify the genes related to intramuscular fat deposition, meat pH, tenderness, and water holding capacity pH^14^. Wan et al.^10^, for example, reported that pork quality deteriorates due to an increase in cortisol levels and high expression of genes related to calcium channel regulation, cell apoptosis, and muscle fiber protein degradation in pigs^10^.

Very few studies have been documented on the relationship between stress and meat quality in animals, and there has been no study in goats on this aspect. The objectives of this study are (1) to determine the differential gene, exon, and isoform expression in *Longissimus dorsi* muscle samples using RNA-seq procedure in Spanish goats subjected to different durations of transportation stress and (2) to determine the differential genes and biological pathways enriched commonly in both blood and muscle to better understand the effects of stress on meat quality in goats.

## Methods

### Animals

This study was conducted in accordance with the ARRIVE guidelines (https://www.nc3rs.org.uk/arrive-guidelines). Prior to beginning of the experiment, the protocols for this research were duly reviewed and approved by the Animal Care and Use Committee at Fort Valley State University. Fifty-four intact male Spanish goats (8-month old; BW = 29.7 ± 2.03 kg), raised primarily on free range pasture with a grain supplement and with ad libitum access to hay and water, were used in this experiment. The animals were randomly subjected to one of three stress treatments (TRT; n = 18 goats/treatment): (1) transported for 30 min (approx. 50 km), (2) transported for 180 min (approx. 200 km), or (3) held in pens (control), on two different days. A livestock trailer (5.0 × 2.3 m; floor space 1.27 m^2^/goat) pulled by a pickup truck was used to transport the goats and the ambient temperatures were − 3.0 ± 1.0 °C and 1.0 ± 1.0 °C on day 1 and 2, respectively. The transported goats were not allowed any recovery time after transport. On both days of the experiment, the same route and distance was used for transporting goats from the respective treatment group. Feed was withheld overnight prior to the day of the experiment.

### Blood sampling

Before and after transportation, blood samples were collected from the jugular vein using disposable needles and vacutainer tubes containing anticoagulant EDTA. The samples were kept on ice until separation of plasma. Blood samples (5 mL) were also collected separately after transportation in PAXgene blood RNA tubes (PreAnalytiX, Qiagen, Germantown, MD) for RNA extraction.

### Catecholamines

Plasma epinephrine and norepinephrine concentrations were determined using the Epinephrine/Norepinephrine ELISA Kit (Abnova, Taipei, Taiwan). Epinephrine and norepinephrine were extracted using a cis-diol-specific affinity gel, acylated, and then converted enzymatically. Quantification of unknown samples was achieved by comparing their absorbances with a standard curve prepared with known standard concentrations. The hormone concentrations were determined following the stepwise procedure and using the reagents and plates provided by the manufacturer. The microtiter plates were read for absorbance values using the Synergy HTX Microplate Reader (Bio-Tek, Winooski, VT).

### Carcass processing

At the slaughter facility, animals were handled and processed according to the Humane Methods of Slaughter Act (https://www.ecfr.gov/current/title-9/chapter-III/subchapter-A/part-313?toc=1). Goats were stunned using a captive bolt pistol and then slaughtered using standard procedures. The *Longissimus dorsi* muscle samples were collected immediately (15 min postmortem) for RNA extraction and glycogen analysis. Muscle samples for RNA extraction and glycogen analysis were wrapped in aluminum foil, quick-frozen in liquid nitrogen, and then transferred to a − 80 °C freezer. The carcasses were cooled at 2 °C for 24 h before processing. After 24 h of storage, each carcass was weighed and then split along the vertebral column into left and right halves. Then 2.5 cm loin/rib chops were collected by slicing using a band saw and vacuum packed for cooking loss and texture analysis. To the extent possible the cuts were made such that the cut surfaces of chops were at right angles to the axes of muscle fibers. Four loin/rib chops were used to measure Commission Internationale de l'eclairage color values (CIE L* a* b*) using a Miniscan XE Plus calorimeter (HunterLab, Reston, VA). The L* values denote the lightness, a* values indicate the redness-green axis, and b* values denote the yellow-blue axis. The instrument was calibrated using a calibration plate before color measurement and the instrument aperture was placed directly on the cut surface for measurement after the chops were allowed to bloom for 40 min. The color values from the four chops were then averaged and recorded. These chops were then vacuum packed and frozen at − 20 °C (1 day of aging). Additional four chops were vacuum packed and aged at 2 °C for 6 days and then frozen at − 20 °C. Later, the chops aged for day 1 and day 6 were used for the determination of Warner Bratzler shear force (WBSF) and cooking loss values. Samples were also collected for calpastatin analysis at 24 h postmortem (1 day of meat aging), troponin-T and desmin analysis at day 1 and day 6 of aging.

### Muscle pH and temperature

Initial pH and temperature were recorded immediately (15 min) after skinning and evisceration and after 24 h of slaughter (final pH) using a portable combination pH meter with a penetrating probe (Pakton Model OKPH1000N, Vernon Hills, IL). The probe was inserted directly into the *Longissimus dorsi* muscle to determine pH values.

### Muscle glycogen

Muscle glycogen concentrations were determined using a Glycogen Assay Kit (Abnova Corporation, Tapei, Taiwan). Duplicate 10 mg tissue samples were homogenized in 200 µL of distilled water and boiled for 10 min to inactivate enzymes. The boiled samples were centrifuged for 10 min at 18,000×*g* to remove insoluble material. The supernatant (10 µL) was collected and final volume of 50 µL/well was adjusted with hydrolysis buffer. After incubation of plates for 30 min at room temperature, 50 µL of reaction mixture was added to each well and plates were incubated again for 30 min at room temperature. Finally, the absorbance was measured at 570 nm using a Synergy HTX Microplate Reader (Bio-Tek, Winooski, VT).

### Cooking loss

The cuts were thawed at 4 °C and placed on aluminum pans and covered with aluminum foil. The cuts were weighed and cooked in a convection oven (Maytag model, Redwood, CA) to an internal temperature of 71 °C. The internal temperature was determined on a representative cut from each pan using thermocouple thermometers (Fisher Scientific, Suwanee, GA). In a randomly chosen cut from each pan, the thermocouple probe was placed in the geometric center of the muscle. The samples were then allowed to come to room temperature before measuring the final weight for assessment of cooking loss percentage^4^.

### Warner–Bratzler shear force

A minimum of three chops were used for shear value determination. Cooked samples were wrapped in aluminum foil and cooled at 4 °C overnight before core removal. Three 1-cm diameter cores were taken parallel to muscle fiber orientation from each chop^4^. The WBSF values were assessed using a TA-XT2 Texture Analyzer (Texture Technologies Corp., Scarsdale, NY), with the samples being sheared at right angles to the orientation of muscle fibers using the Warner–Bratzler shear attachment (Texture Technologies Corp., Scarsdale, NY). The instrument was set with a 25-kg load cell and crosshead speed of 200 mm/min.

### Calpastatin, desmin and troponin-T

Muscle calpastatin (CAST) levels were determined using a CAST ELISA kit (MyBioSource, San Diego, CA). The CAST ELISA kit applies the competitive enzyme immunoassay technique utilizing a polyclonal anti-CAST antibody and CAST-horseradish peroxidase (HRP) conjugate. A standard curve was plotted relating the intensity of the color (OD) to the concentration of standards. The CAST concentration in each sample was interpolated from this standard curve. Muscle desmin (DES) concentration was determined using a DES ELISA kit (MyBioSource, San Diego, CA). The DES ELISA kit applies the competitive enzyme immunoassay technique utilizing a polyclonal anti-DES antibody and a DES-HRP conjugate. The DES concentration in each sample was interpolated from this standard curve. Muscle troponin-T concentration was determined using Goat Troponin-T ELISA kit (MyBioSource, San Diego, CA). This kit utilizes the double antibody ELISA technique. In this method, the color intensity and quantity of the target analyte in the sample are positively correlated.

### RNA extraction

Muscle RNA was extracted from the *Longissimus dorsi* muscle samples collected at 15 min postmortem and after 6 days of aging loin/rib chops using RNeasy Fibrous Tissue Mini Kit (Qiagen, Germantown, MD) according to manufacturer’s procedures. Muscle tissue samples (30 mg) were first lysed and then homogenized in 300 µL of RLT Buffer in each 2 mL RNase free microcentrifuge tube. Then, 590 µL of RNase-free water and 10 µL of protein K were added, mixed, and incubated at 55 °C for 10 min. To the lysate, 350 µL of 100% ethanol was added to provide ideal binding conditions. Lysate was then loaded onto the RNeasy Mini column (2 mL collection tube) and RNA was allowed to bind to the silica membrane, and all contaminants were efficiently washed away. The residual amounts of DNA remaining were removed by adding 10 µL of DNase stock solution. To the RNeasy Mini column, 350 µL of RW1 buffer was added, centrifuged at 8000×*g* for 15 s and the flow through was discarded. To this, 500 µL of RPE buffer was added, centrifuged for 15 s, and again for 2 min at 8000×*g*. Finally, 50 µL of RNase free water was added to RNase column in new 1.5 mL RNase free tubes and centrifuged at 8000×*g* for 1 min to get purified concentrated RNA. The samples were stored at − 80 °C freezer until shipped for sequencing and bioinformatics analysis. Muscle samples from 27 animals (n = 9 goats/TRT) were collected at both 15 min and 6 days postmortem were used for RNA extraction giving a total of 54 muscle samples. Blood samples from these 27 goats (n = 9 goats/TRT) were also used for RNA extraction. Blood RNA was extracted using a MagMAX Stabilized Blood Tubes RNA Isolation Kit by applied biosystems (Thermo Fisher Scientific, Waltham, MA) according to manufacturer’s instructions.

### RNA-Seq library preparation and sequencing

The purity of RNA was checked using a NanoDrop spectrophotometer (Implen, CA, USA). The RNA degradation and contamination were monitored on 1% agarose gels. The RNA integrity and quantification were assessed using RNA Nano 6000 Assay Kit of the Bioanalyzer 2100 system (Agilent Technologies, CA, USA). The samples were then sent to Novogene Corporation Inc. (Sacramento, CA, USA) for mRNA isolation, RNA-seq library preparation, and sequencing procedures.

### Quality control

After processing the raw data (raw reads) of FASTQ format through fastp, the clean data (clean reads) were obtained by removing reads with low quality and reads containing adapter and poly-N sequences (N > 10%). From the clean reads, the proportions of bases with a phred base quality score of Q20 and Q30, and the proportion of G and C base numbers of the total bases were calculated. Clean data were used for all downstream analyses.

### Reads mapping on the caprine reference genome and gene expression analysis

Reads were mapped directly to transcriptome for the significant expression of differentially expressed genes. All reference genome and gene annotation files were downloaded directly from the genome website browser (NCBI/UCSC/Ensembl). Clean paired-end reads were mapped directly to the reference genome of goat (*Capra hircus*) using the software HISAT2^15^, which is available at https://registry.opendata.aws/jhu-indexes/. StringTie software^16^, which is freely available at http://ccb.jhu.edu/software/stringtie/index.shtml?t=manual, was used to identify novel genes and transcripts. Gffcompare was used to compare the StringTie assemblies to the annotation files that helped to sort out the new genes from the known ones. Gene expression levels were estimated by the transcript abundance that mapped to genome or exon. Transcript abundances were estimated as reads per kilobase of exon model per million mapped reads (RPKM), which was calculated based on the length of the gene and reads count mapped to the genes.

### Statistical analysis

Blood hormone and metabolite data were analyzed using General Models Procedure in SAS (release 9.1, SAS Institute, Cary, NC, USA) with TRT and day as independent variables. The meat quality characteristics were analyzed using PROC MIXED procedure in SAS with TRT and day as fixed effects and animal as the random effect. Aging time effect was analyzed using a repeated statement in the mixed model analysis. When significant by ANOVA at *P* < 0.05, the means were separated using the LSD test. Pearson correlation analysis of muscle pH, temperature, cooking loss, WBSF, calpastatin, troponin-T, and desmin variables was conducted using the PROC CORR procedure in SAS. Bioinformatics analysis of RNA-seq results was performed by Novogene Corporation, Inc. While RNA-seq results were obtained for all 54 muscle samples, sequencing results were obtained only for 18 out of 27 blood samples sent for analysis, which resulted in unbalanced biological replicates for blood analysis (n = 7 for control, 4 for 30 min, 7 for 180 min). Alternative splicing (AS) analysis was performed using the software rMATS^17^. The rMATS software and user manual are freely available for download at https://rnaseq-mats.sourceforge.net/. Differential gene expression analyses for both muscle and blood samples were carried out using DESeq2^18^. Hierarchical clustering was used to sort out the genes with similar expression patterns under different experimental conditions. In addition to the fragments per kilobase of transcript per million fragments mapped (FPKM) cluster, the H-Cluster was used to cluster the log2 (fpkm + 1) for gene expression pattern. The resulting *P* values were adjusted using Benjamini and Hochberg’s approach for controlling false discovery rate (FDR)^19^. The fold changes (in log2 scale), *P* values and *q*-values (corrected *P* values) of differentially expressed genes (DEG) were recorded from DESeq2 output. Genes with a q value of < 0.05 were considered to be significantly expressed, which gave an FDR of 5% or less. Gene Ontology (GO) enrichment analysis was executed by the ClusterProfiler R package, which corrected the gene length bias. Statistical enrichment of differentially expressed genes in KEGG (Kyoto Encyclopedia of Genes and Genomes) pathway was analyzed by ClusterProfiler R package.

### Ethics approval and consent to participate

The methods in this investigation were conducted according to relevant guidelines and regulations. The animal care protocols were approved by the FVSU Agricultural and Laboratory Animal Care and Use Committee prior to beginning of the study following the ADSA-ASAS-PSA Guide for Care and Use of Agricultural Animals in Research and Teaching.

## Results

### Blood hormone concentrations

**Figure 1:**
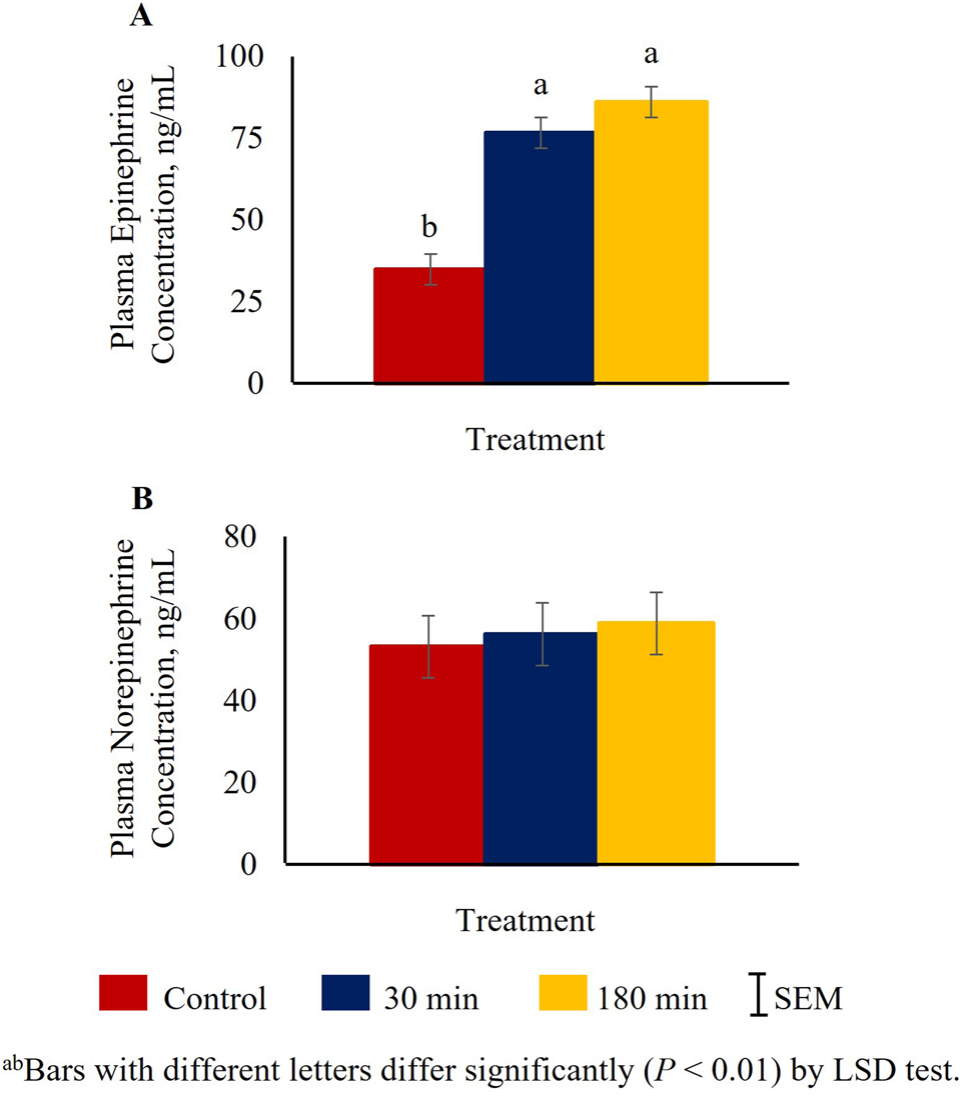
Effects of stress treatment (Control = not transported but held in pens; 30 min = transported for 30 min; 180 min = transported for 180 min) on mean ± SEM plasma (**A**) epinephrine and (**B**) norepinephrine concentrations in goats.

Plasma epinephrine concentrations were higher (*P* < 0.01) in both 180 min and 30 min groups compared to the control group (Fig. 1A). However, plasma norepinephrine concentrations were not significantly affected by TRT (Fig. 1B).

### Carcass and meat quality characteristics

**Figure 2:**
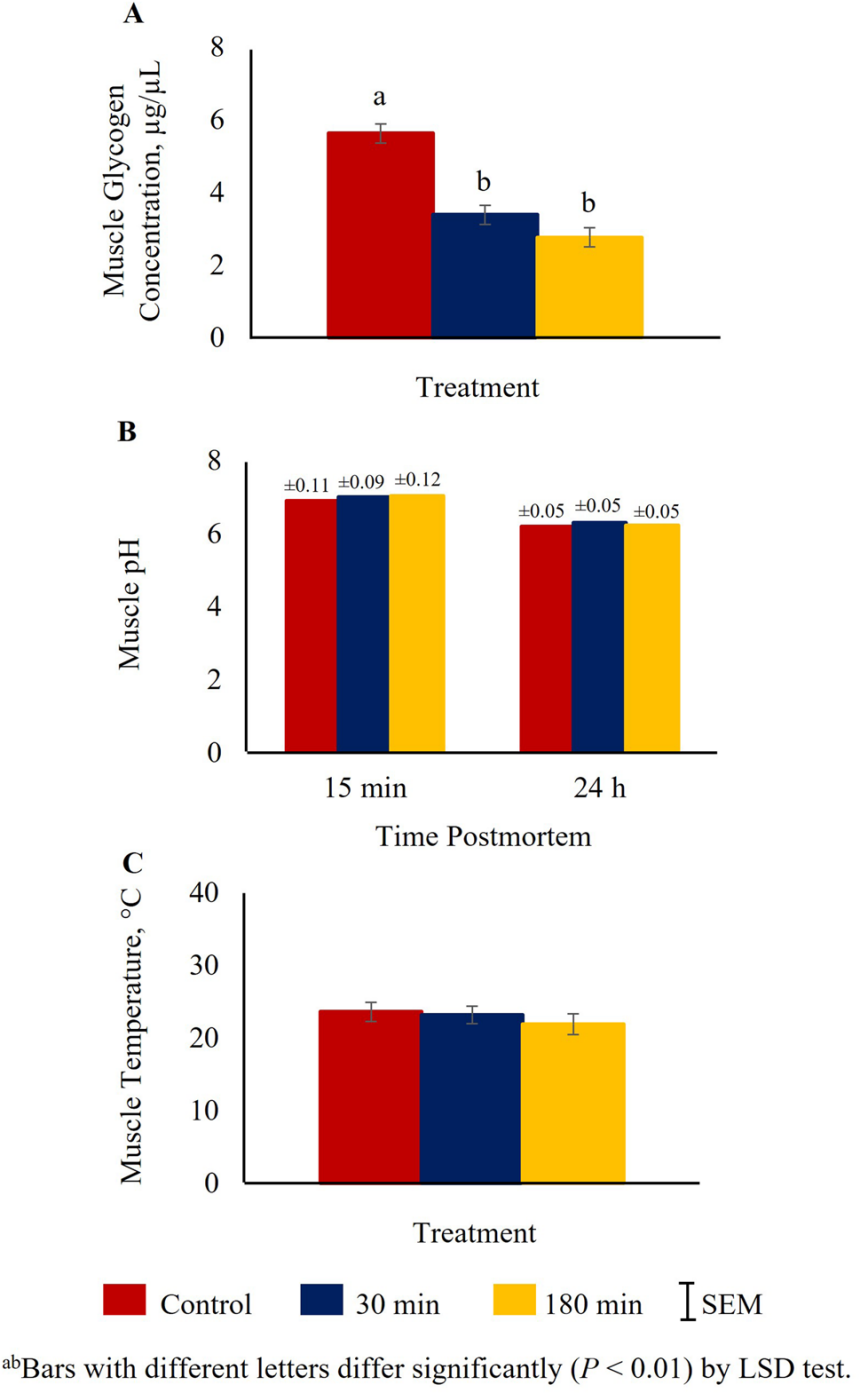
Effects of stress treatment (Control = not transported but held in pens; 30 min = transported for 30 min; 180 min = transported for 180 min) on mean ± SEM *Longissimus dorsi* muscle (**A**) glycogen concentration and (**B**) initial (15 min postmortem) and final (24 h postmortem) pH and (**C**) temperature (15 min postmortem) values in goats.

Hot and cold carcass weights and live weights were not significantly affected by TRT (Supplementary Fig. S1). Glycogen concentrations measured at 15 min postmortem were significantly affected by the treatment (*P* < 0.01). The concentrations were higher in the control group compared to 30 min or 180 min TRT groups (Fig. 2A). *Longissimus dorsi* muscle pH values measured at 15 min and 24 h and temperature measured at 15 min postmortem were not significantly affected by TRT (Fig. 2B,C). Among the color values (L*, a*, b*) of loin/rib chops (*L. dorsi*) determined at 24 h postmortem, only L* values were influenced by TRT (Supplementary Fig. S2), with the 180 min TRT group having the lowest (*P* < 0.01) L* values.

**Figure 3:**
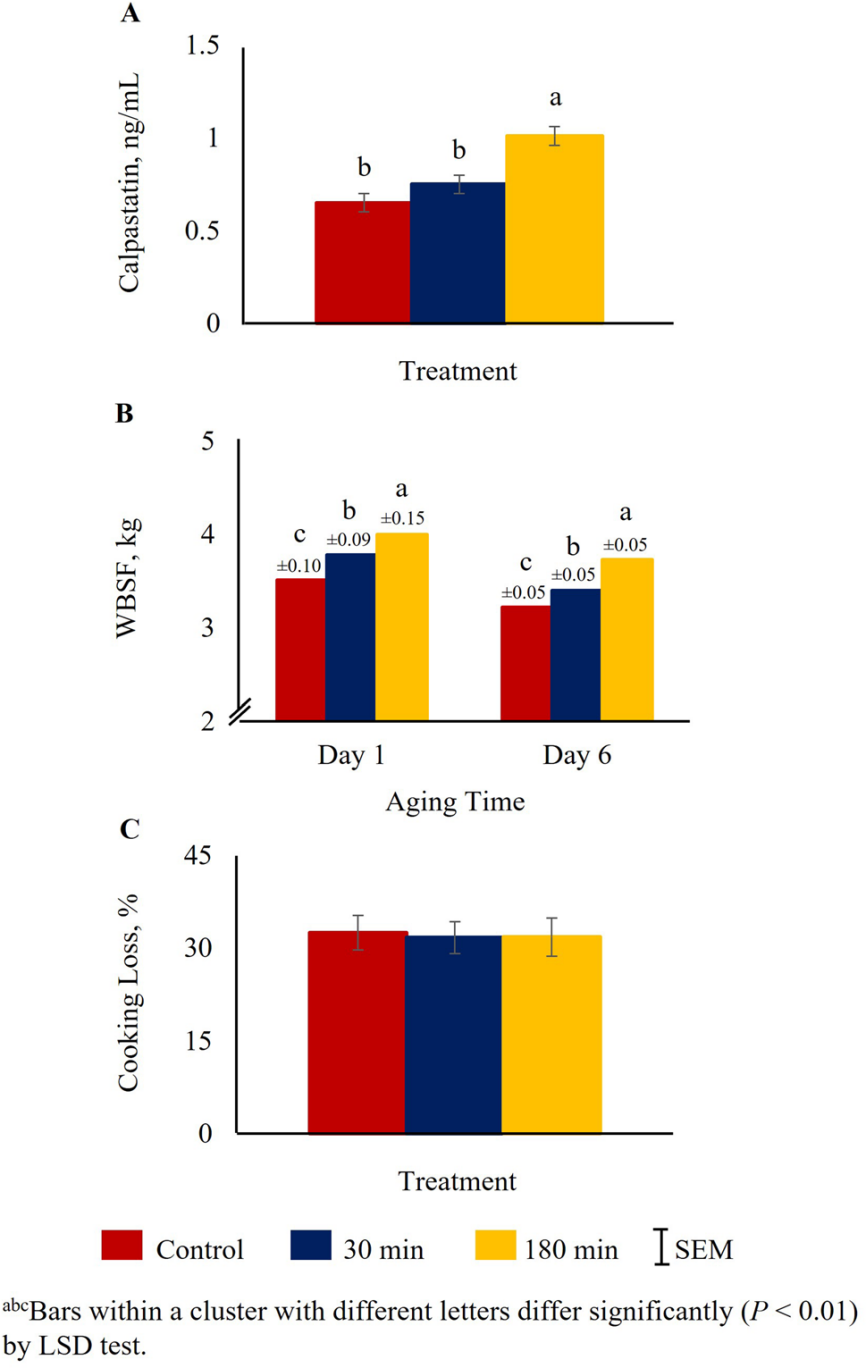
Effects of stress treatment (Control = not transported but held in pens; 30 min = transported for 30 min; 180 min = transported for 180 min) on mean ± SEM loin/rib (*Longissimus dorsi*) chop (**A**) calpastatin activity (24 h postmortem), (**B**) Warner–Bratzler shear force value (day 1 and day 6 of aging), and (**C**) cooking loss percentage (24 h postmortem) in goats.

Muscle calpastatin values (24 h) were higher (*P* < 0.01) in the 180 min group compared to the 30 min or control TRT groups, and there was no significant difference between the control and 30 min groups (Fig. 3A). The WBSF values at day 1 and day 6 were low in the control group and increased with increasing transportation time. At both aging times, the WBSF values were the highest in 180 min, lowest in the control group and intermediate in 30 min TRT group (*P* < 0.01; Fig. 3B). However, TRT did not have a significant effect on cooking loss of loin/rib chops measured at 24 h postmortem (Fig. 3C).

**Figure 4:**
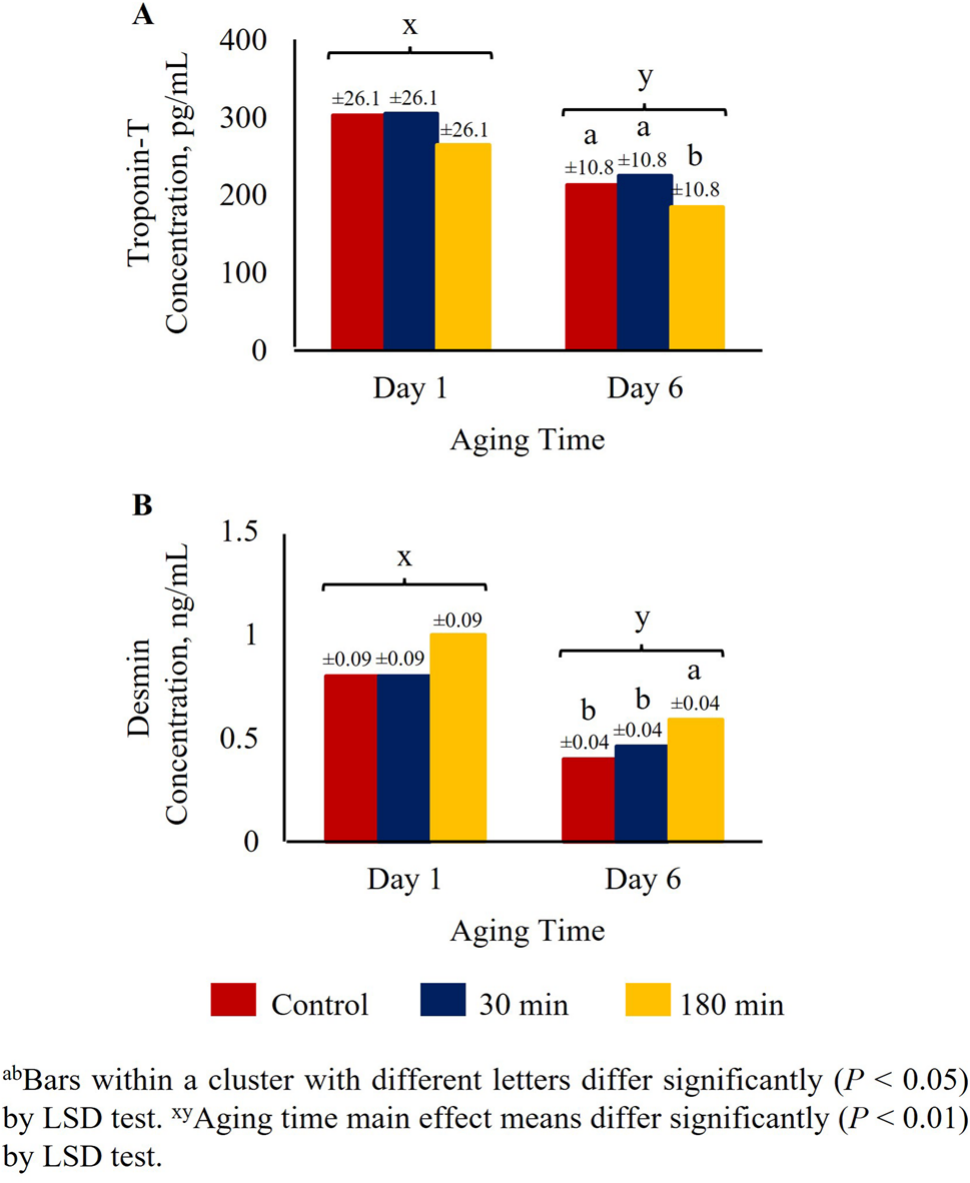
Effects of stress treatment (Control = not transported but held in pens; 30 min = transported for 30 min; 180 min = transported for 180 min) on mean ± SEM loin/rib (*Longissimus dorsi*) chop (**A**) troponin-T and (**B**) desmin concentrations determined at day 1 and day 6 of aging in goats.

Troponin-T levels in day 1 meat samples (*Longissimus dorsi*) were not affected by TRT; however, at day 6, troponin-T was lower in 180 min group compared to 30 min or control groups (*P* < 0.05; Fig. 4A). The main effect of aging time was significant, with the overall troponin-T level lower at day 6 compared to day 1 (*P* < 0.01). Desmin levels in day 1 meat samples (*Longissimus dorsi)* were not affected by TRT; however, at day 6, desmin was higher in the 180 min group compared to 30 min or control group (*P* < 0.05; Fig. 4B). The main effect of aging time was significant, with the overall desmin level lower at day 6 compared to day 1 (*P* < 0.01).

### Correlations among tenderness-related variables

Pearson correlation coefficients among the meat quality variables are presented in Table 1. Initial pH of *L. dorsi* was negatively correlated with temperature measured at 15 min postmortem (*P* < 0.05). There were significant positive correlations among muscle desmin level after 6 days of aging and pH at 24 h (*P* < 0.05), calpastatin at day 1 (*P* < 0.05), and WBSF at day 1 (*P* < 0.01). There was also a significant negative correlation between muscle temperature at 15 min postmortem and desmin concentration at day 6 (*P* < 0.05). Also, calpastatin activity at day 1 and WBSF at day 1 were significantly positively correlated (*P* < 0.05).

**Table 1:** Pearson correlation coefficients among the tenderness-related quality variables of loin/rib chops (*Longissimus dorsi*) from carcasses of goats subjected to transportation stress.

Variables	Final pH (24 h)	Temp (15 min)	Calpastatin day 1	WBSF day 1	WBSF day 6	Troponin-T day 1	Troponin-T day 6	Desmin day 1	Desmin day 6	CL^d^ day 1
Initial pH (15 min)	− 0.048	− 0.313	0.234	0.077	0.154	− 0.177	0.120	− 0.177	0.074	− 0.121
	*0.729*	*0.021*	*0.088*	*0.579*	*0.267*	*0.200*	*0.386*	*0.202*	*0.593*	*0.382*
Final pH (24 h)		− 0.113	0.144	0.138	− 0.120	0.010	0.021	0.106	0.272	0.030
		*0.415*	*0.300*	*0.321*	*0.388*	*0.943*	*0.879*	*0.448*	*0.047*	*0.832*
Temp^a^ (15 min)			− 0.178	− 0.171	− 0.112	− 0.102	− 0.116	− 0.129	− 0.313	− 0.072
			*0.197*	*0.218*	*0.419*	*0.464*	*0.405*	*0.354*	*0.021*	*0.607*
Calpastatin Day 1^b^				0.292	0.252	− 0.125	− 0.161	0.124	0.332	− 0.122
				*0.032*	*0.066*	*0.369*	*0.245*	*0.372*	*0.014*	0.381
WBSF Day 1					0.611	− 0.104	− 0.058	0.141	0.404	0.137
					< *0.001*	*0.453*	*0.679*	*0.307*	*0.002*	*0.325*
WBSF Day 6^c^						− 0.060	− 0.122	0.086	0.228	− 0.139
						*0.666*	*0.379*	*0.537*	*0.098*	*0.318*
Troponin-T Day 1							0.105	0.377	0.214	0.259
							*0.448*	*0.005*	*0.121*	*0.059*
Troponin-T Day 6								− 0.125	− 0.035	0.169
								*0.369*	*0.800*	*0.221*
Desmin Day 1									0.628	0.105
									< *0.001*	*0.456*
Desmin Day 6										0.132
										*0.343*

^a^Muscle temperature; ^b^24 h postmortem; ^c^6 days postmortem; ^d^precent cooking loss; numbers in italics are probability values.

### Sequencing

A total of 72 cDNA libraries were constructed using blood samples and *Longissimus dorsi* tissue samples. For 27 tissue samples of 15 min postmortem *Longissimus dorsi*, an average of 33,883,840, 35,441,056, and 34,220,284 raw reads were obtained for control, 30 min, and 180 min TRT groups, respectively, and 33,196,253, 34,870,088, and 33,589,206 clean reads were obtained with phred base quality scores of Q20 > 97%, Q30 > 93%, and an average GC content > 51% (Table 2). For day 6, 27 tissue samples of *Longissimus dorsi* generated an average of 32,708,893, 34,884,749 and 33,729,003 raw reads for control, 30 min, 180 min TRT groups, respectively, and 32,183,347, 34,295,096 and 33,132,906 clean reads with phred base quality scores of Q20 > 97%, Q30 > 92%, and an average GC content > 50%.

**Table 2:** Summary of sequencing results of *Longissimus dorsi* muscle samples from carcasses of goats subjected to transportation stress treatment (TRT).

TRT	PM time^d^	Raw reads	Clean reads	Unique reads	Total mapped reads	Total mapping %	Q20^e^ %	Q30^f^%	GC content^g^%
Control^a^	15 min	33,883,840	33,196,253	56,726,416	64,874,267	97.71	97.90	93.76	51.87
Control	6 days	32,708,893	32,183,347	51,274,084	62,616,886	97.29	97.42	92.41	50.25
30 min^b^	15 min	35,441,056	34,870,088	61,413,669	68,121,055	97.68	97.96	93.98	52.56
30 min	6 days	34,884,749	34,295,096	55,929,162	66,684,959	97.22	97.33	92.24	50.86
180 min^c^	15 min	34,220,284	33,589,206	58,596,054	65,605,122	97.65	97.84	93.69	52.73
180 min	6 days	33,729,003	33,132,906	54,265,860	64,433,338	97.24	97.48	92.58	51.13

^a^Not transported but held in pens; ^b^transported for 30 min; ^c^transported for 180 min; ^d^postmortem (PM) sample collection time (15 min or after 6 days of aging); ^e^phred values greater than 20 base number contain the percentage of total bases; ^f^phred values greater than 30 base number contain the percentage of total bases; ^g^the percentage of guanine and cytosine base numbers of total bases.

### Gene expression

The result of the principal component analysis (PCA) of gene expressions in blood and meat samples from different groups (TRT and meat aging time) is shown in Supplementary Fig. S3. The PCA 1 explains 73% of the total variation in gene expressions and PCA 2 explains 8.55% of the total variation in gene expressions. There was a clear differentiation between blood and tissue samples; however, there were no different clusters based on TRT or sample collection time. The summaries of FPKM of *Longissimus dorsi* samples are given in Supplementary Table T1. For blood and muscle samples from all treatment groups, the FPKM density showed similar skewed distribution with approximately 60.29–64.36% of genes being lowly expressed (FPKM 0–1; Supplementary Fig. S4). The summary of genes expressed in muscle samples from each treatment group and genes co-expressed are given in Supplementary Fig. S5. For comparisons among the TRT groups for 15 min postmortem muscle samples, there were 10,294 genes co-expressed, and for 6 days, there were 10,489 genes co-expressed. Our presentation of results of the differential gene expressions, gene ontology and KEGG pathways in this paper will primarily focus on muscle samples and to a limited extent on blood samples when DEGs and pathways were commonly enriched in both blood and muscle samples.

### Differential gene expression

**Figure 5:**
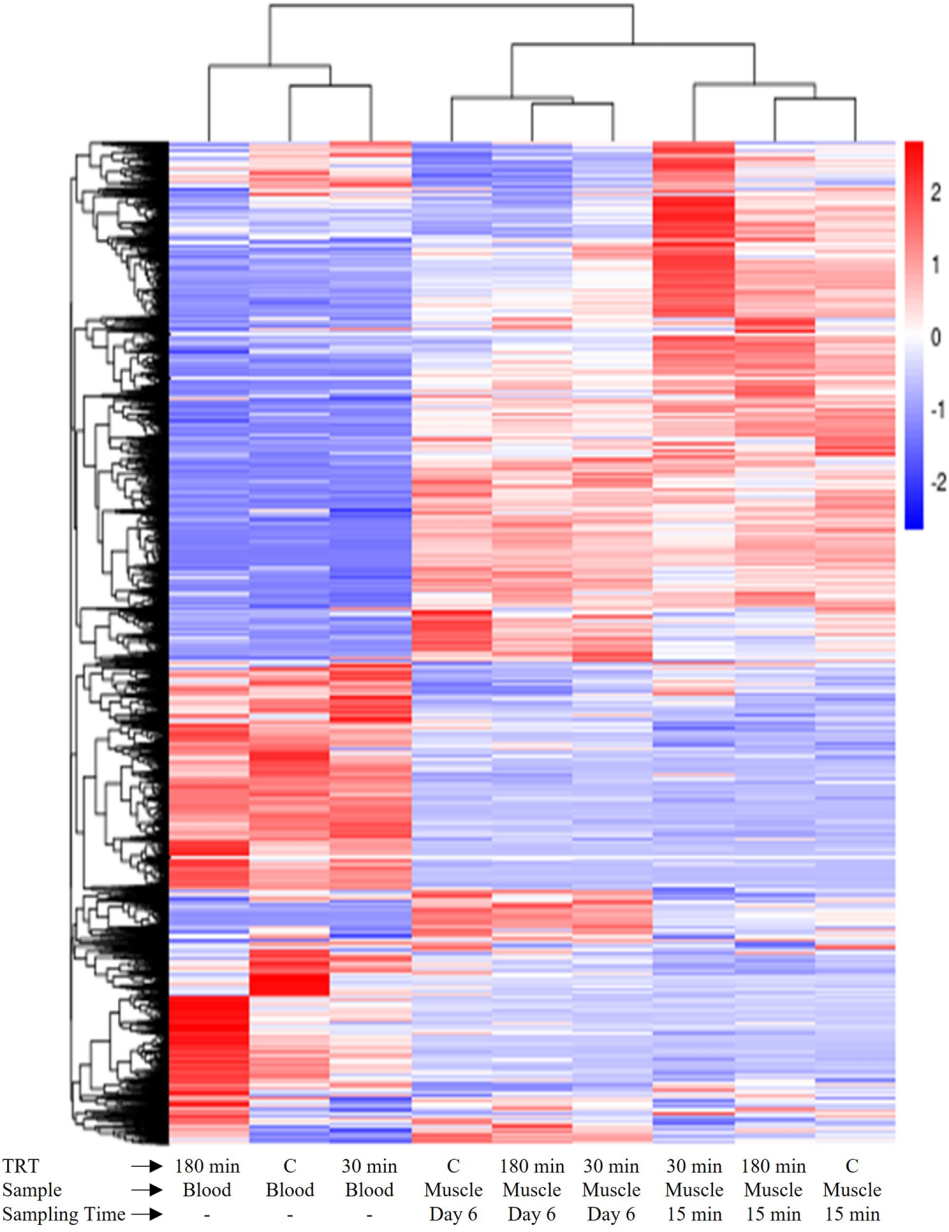
Heat map of blood samples from goats subjected to stress treatments (TRT: control, C = not transported but held in pens; 30 min = transported for 30 min; or 180 min = transported for 180 min) and of meat samples (*Longissimus dorsi*) at 15 min postmortem or after 6 days.

**Figure 6:**
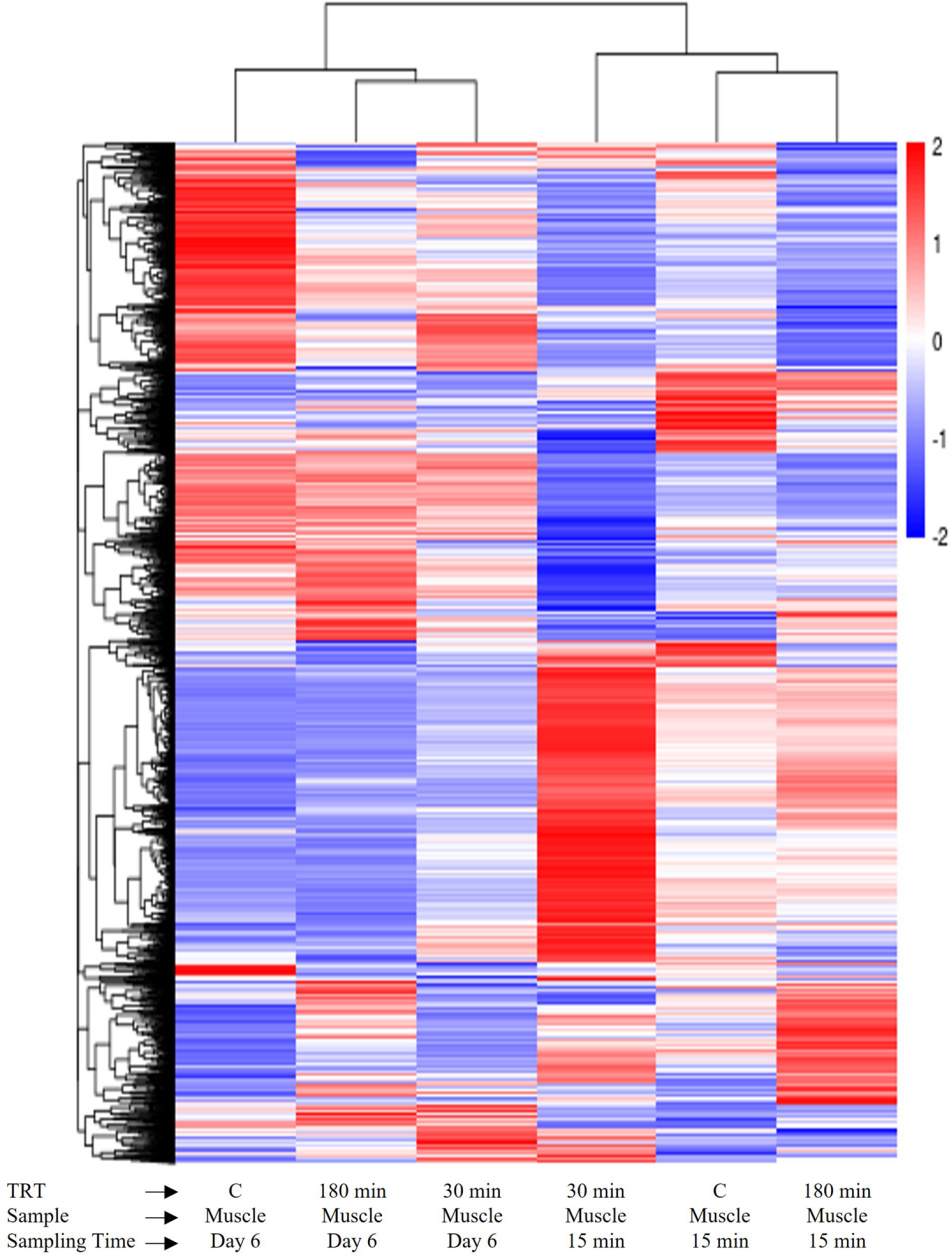
Heat map of meat samples (*Longissimus dorsi*) at 15 min postmortem or after 6 days from goats subjected to stress treatments (TRT: control, C = not transported but held in pens; 30 min = transported for 30 min; or 180 min = transported for 180 min).

The total, upregulated, and downregulated DEG counts in blood samples for TRT comparisons and for muscle samples for TRT comparisons at each postmortem time are presented in Supplementary Fig. S6. Hierarchical clustering of DEGs in both blood and muscle samples for different TRT and postmortem time groups were visualized by means of a heat map (Fig. 5). A similar heat map was also created separately for muscle samples (Fig. 6) to visualize the hierarchical clustering patterns. The blue colored lines identify a lower level of gene expression (between 0 and − 2), while the red colored lines indicate the higher level of gene expression (between 0 and 2).

The DESeq2 software identified a total of 4125 that were differentially expressed for 15 min muscle samples in 30 min vs. control comparison, of which 2023 genes, such as CASP7, FOXO3, PTX3, G3BP2, CAMK2D were upregulated with gene activities related to caspase 7, forkhead box O3, pentraxin 3, G3BP stress granule assembly factor 2, and calcium/calmodulin dependent protein kinase II delta, and 2102 genes such as VSIR, COPRS, STARD5, RIPK1, and SLC12A7 were downregulated (Supplementary Table T2; Supplementary Fig. S7). The day 6 muscle samples in 30 min vs. control comparison showed that 1252 genes were found differentially expressed of which 611 DEGs were upregulated with significant expression genes such as CASP7, FOXO3, HSPA12A, OXSR1, PRKAA2, with gene functions related to caspase 7, forkhead box O3, heat shock protein family A member 12A, oxidative stress response 1, and protein kinase AMP-activated catalytic subunit alpha 2, while 641 DEGs were found downregulated with significant enrichment of genes such as MDK, MYCBPAP, NTN5, LXN, and LTB with gene functions related to midkine, MYCBPAP associated protein, Netrin 5, latexin, and lymphotoxin beta (Supplementary Table T2; Supplementary Fig. S7).

The 15 min muscle samples in 180 min vs. control comparison revealed that 954 genes such as FOXO1, CASP7, PFKFB4, CFLAR, and CDK2 were upregulated that are related to activities of forkhead box O3, Caspase 7, 6-phosphofructo-2-kinase/fructose-2,6-biphosphatase 4, CASP8 and FADD like apoptosis regulator, and cyclin-dependent kinase 2, and 1007 DEG were downregulated with significant expression of genes such as, RASSF4, SCN2B, PSAT1, ZNF827, and KY that are related to the gene activities related to Ras association domain family member 4, sodium voltage-gated channel beta subunit 2, phosphoserine aminotransferase 1, zinc finger protein 827 and kyphoscoliosis peptidase (Supplementary Table T3; Supplementary Fig. S7). In 180 min vs. control comparison, the day 6 samples showed that 738 DEGs were upregulated such as BAG2, FOXO1, CASP7, GADD45G, and WNT5B that are related to functions such as BCL2 associated athanogene 2, forkhead box O1, caspase 7, growth arrest and DNA damage inducible gamma, and Wnt family member 5B, and 880 DEGs were down regulated such as FAM110B, VEGFC, CCL2, ADAMTS13, DAGLA that regulate gene functions related to family with sequence similarity 110 member B, vascular endothelial growth factor C, C–C motif chemokine ligand 2, ADAM metallopeptidase with thrombospondin type 1 motif 13, and diacylglycerol lipase alpha (Supplementary Table T3; Supplementary Fig. S7).

The DESeq2 software identified a total of 430 DEGs in blood samples for control vs. 30 min comparison, of which 303 genes were downregulated and 127 genes were upregulated. For 180 min vs. control comparison of blood samples, 741 DEGs were upregulated and 506 DEGs were downregulated. The CASP14 gene, among several caspases, was significantly upregulated when treatments were compared. Caspases were also significantly upregulated in muscle samples in our study. In addition, PTX3 and GADD45 were significantly upregulated in both blood and muscle samples in our treatment comparisons.

### Gene ontology

A total of 10,633 DEGs were annotated for the GO terms: biological processes (BP), cellular components (CC), and molecular functions (MF) for six different treatment comparisons of blood samples and 15 min and day 6 muscle samples. Significantly upregulated biological process terms in comparison of muscle samples from treatments include proteolysis involved in cellular protein catabolic process, response to endoplasmic reticulum stress, cellular response to insulin stimulus, regulation of apoptotic signaling pathway, and cellular response to corticosteroid stimulus among other processes. The upregulated genes in both blood and meat samples include PTPN1, HYAL2, SOCS and KLF. Biological processes that were downregulated in comparison of muscle samples from treatments include regulation of immune response, regulation of cytokine production, regulation of leukocyte proliferation, T cell differentiation, and negative regulation of cytoskeletal organization (Supplementary Table T4).

### KEGG

**Figure 7:**
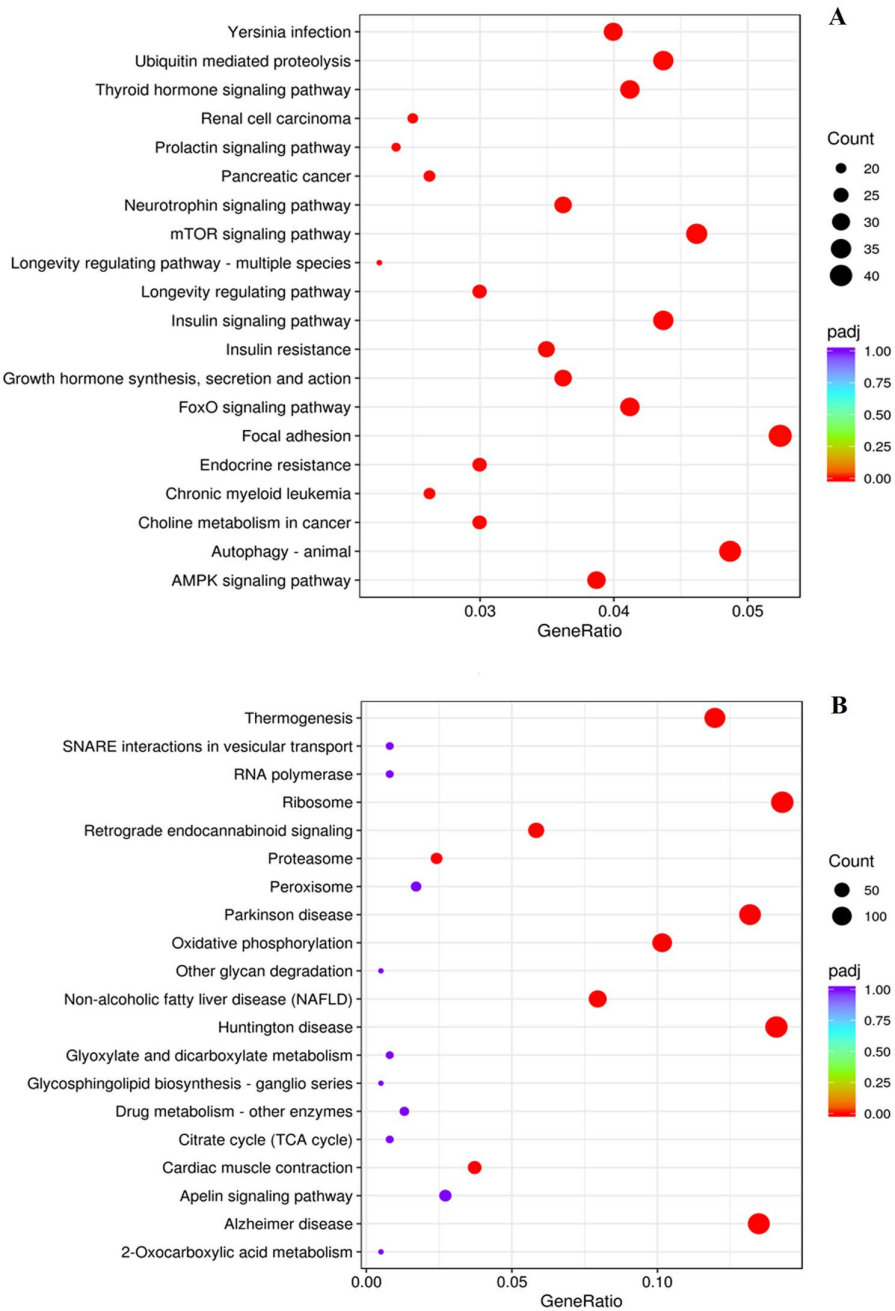
(**A**) Upregulated and (**B**) downregulated KEGG pathways in meat (*Longissimus dorsi*) samples at 15 min postmortem, from goats subjected to control (not transported but held in pens) vs. 30 min (transported for 30 min) stress treatments.

**Figure 8:**
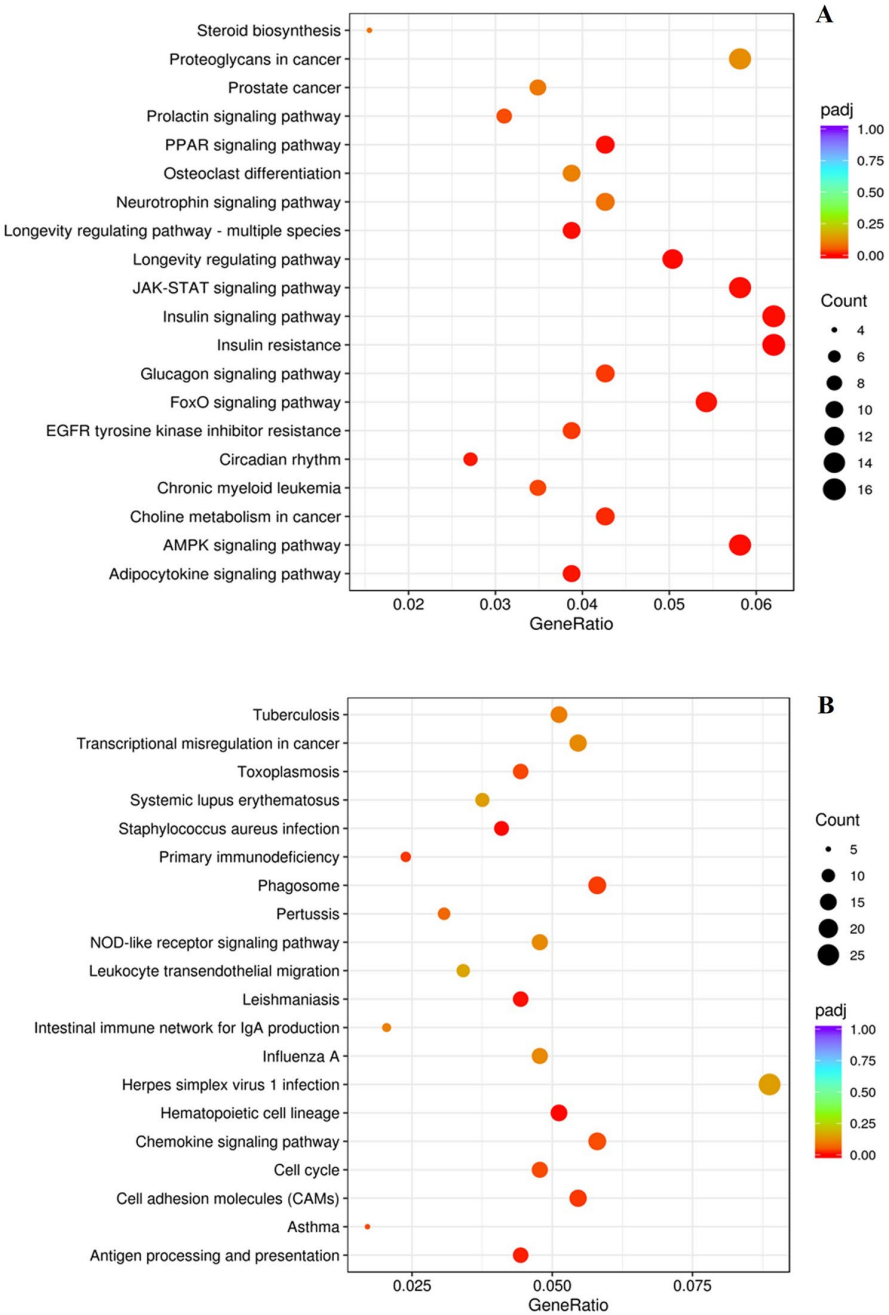
(**A**) Upregulated and (**B**) downregulated KEGG pathways in meat (*Longissimus dorsi*) samples after 6 days, from goats subjected to control (not transported but held in pens) vs.30 min (transported for 30 min) stress treatments.

All DEGs obtained from DESeq2 method were mapped to KEGG pathways to determine genes and pathways related to stress and its effect on meat quality. For 30 min vs. control comparison of muscle samples collected at 15 min postmortem, significantly enriched (*q* < 0.1) upregulated pathways were FoxO signaling pathway, AMPK signaling pathway, insulin signaling pathway, mTOR signaling pathway, and ubiquitin mediated proteolysis (Fig. 7A), and the downregulated pathways were oxidative phosphorylation, ribosome, citric acid cycle, and glyoxylate and dicarboxylate metabolism (Fig. 7B). The upregulated pathways for 30 min vs. control comparison of muscle samples at 6 days were glucagon signaling pathway, JAK-STAT signaling pathway, PPAR signaling pathway, FOXO signaling pathway, AMPK signaling pathway, and insulin signaling pathway (Fig. 8A) and the downregulated pathways were chemokine signaling pathways, NOD-like receptor signaling pathway, antigen processing and presentation, and leukocyte transendothelial migration (Fig. 8B). Autophagy was among the top 20 upregulated pathways in both blood and muscle samples.

For 180 min vs. control muscle samples at 15 min postmortem, the upregulated pathways were RNA transport, ribosome biogenesis, protein processing in endoplasmic reticulum, p53 signaling pathway, glucagon signaling pathway, and fructose and mannose metabolism, and the downregulated pathways were Wnt signaling pathway, Ras signaling pathway, NF-kappa B signaling pathway, cAMP signaling pathway, and calcium signaling pathway (Supplementary Fig. S8). The upregulated pathways for 180 min vs. control comparison of muscle samples at 6 days were RNA transport, protein processing in endoplasmic reticulum, JAK-STAT signaling pathway, arginine and proline metabolism, aminoacyl- tRNA biosynthesis, and proteasome, and the downregulated pathways were cell adhesion molecules (CAM), complement and coagulation cascades, regulation of lipolysis in adipocytes, Th1 and Th2 cell differentiation, and ECM-receptor interaction (Supplementary Fig. S9).

## Discussion

Societal awareness regarding handling of livestock prior to processing on welfare aspects and product quality characteristics has increased in the recent years. Transportation of animals from the point of production to the packing plant is a critical step that involves multiple stressors that could negatively impact the animal’s emotional and physiological status that, if mismanaged, could result in compromised animal wellbeing and consequently in downgraded carcasses, lowered meat quality, reduced economic returns, and potentially lost market share. The absence of oxygen supply after exsanguination requires muscles to switch to anaerobic metabolism^7^. The extent of postmortem anaerobic glycolysis depends on the antemortem stress level and glycogen depletion triggered by epinephrine. Plasma epinephrine concentrations were higher in the 30 min and 180 min groups compared to the control group. Stress stimulates the hypothalamus–pituitary–adrenal (HPA) axis and sympatho-adrenal axis that activate the adrenal gland to release stress hormones. Catecholamines are released into the blood during stress, which activate glycogenolysis, resulting in increases in blood glucose and muscle lactic acid concentrations^3^. During transportation, contracting muscles need a continuous supply of ATP, which is provided by the glycolytic pathway^20^. In healthy rested animals, postmortem muscle glycogen levels are high, while stressed animals have lower levels of glycogen^21^. In our study, glycogen levels determined at 15 min postmortem were higher in the control group compared to the transported groups. This is consistent with the results of previous studies on stress in goats. For example, Alpine goats subjected to a short-term stress had lower *longissimus* muscle glycogen concentration at 15 min postmortem compared to non-transported goats^3^. The extend and rate of postmortem glycolysis determine to a large extent the quality characteristics of meat such as tenderness, color, and juiciness^2^.

Decreased glycolytic potential of muscles can result in darker colored meat in small ruminants^3^ that is reflected by lower L* values. The incidence of dark cutting meat is influenced by various factors, including preslaughter handling methods, that could also influence the water holding capacity of meat. Handling stress prior to processing can induce oxidative stress, which is one of the main causes of meat quality deterioration besides microbial spoilage^7^. Oxidative stress occurs when the antioxidant defense mechanisms in the body is not able to copy up with the rate of free radical production because of stress^7^. Lipid oxidation and muscle pigment oxidation rate affect color and other quality characteristics of meat and stress can accelerate oxidation of meat due to its higher polyunsaturated content^22^. However, color values or cooking loss percentages were not affected by TRT in the current study.

Shear values of meat were the highest in 180 min group, lowest in the control group, and intermediate in the 30 min group, which indicates that the extent of development of meat tenderness corresponds with the intensity and/or duration of stress. Intramuscular fat is an important trait that can affect the tenderness of meat; however, goat meat has very little marbling, if any. Therefore, this study did not focus on intramuscular fat content as this variable is not of major importance in goat meat. Goats also have very little subcutaneous fat unlike beef and lamb. The fat content is lower, and the fatty acid profile is healthier in goat meat compared to beef and lamb, which is attractive to health-conscious consumers^23^. The lack of adequate subcutaneous fat and smaller carcass sizes make goat carcasses prone to cold shortening^24^. Meat tenderness development can depend on sarcomere shortening and reduced proteolytic activity, both being caused by the same postmortem conditions^8^. It is not clear if cold shortening occurred in the present study since we did not measure hourly pH and temperature and sarcomere length.

Other factors that could affect goat meat tenderness are higher calpastatin activity^25^ and the oxidation rate^4,22^, which can interfere with calpain activity as these proteolytic enzymes need reducing conditions for optimum activity^26^. While troponin-T appeared to have degraded after 6 days of aging in the 180 min group, desmin levels remained higher in the 180 min group after 6 days than the other TRT groups. This suggests an interrelationship among calpastatin activity, desmin degradation, and WBSF values, all of which are dictated by the postmortem environment in muscles caused by preslaughter stress. Gadiyaram et al.^24^ observed a tendency for desmin degradation during postmortem aging that corresponded with decrease in WBSF values in goat meat. Desmin is one of the cytoskeletal proteins that maintains structural integrity of skeletal muscle fiber. In the present study, there were significant positive correlations among muscle desmin level after 6 days of aging, and ultimate pH, calpastatin activity at day 1, and WBSF values at day 1. In skeletal muscles, calpastatin has four calpain-inhibiting domains^7^. Calpastatin activity postmortem could have influenced desmin degradation in the present study as it inhibits µ-calpain activity in a pH-dependent fashion and influences both the rate and extent of meat tenderization^8^.

Preslaughter stress in goats significantly changed energy metabolism early postmortem, which influences tenderness development during aging. Gene ontology terms showed that several biological processes related to energy metabolism were upregulated. The AMPK pathways were enriched in the transported group compared to the control group of goats, which is crucial for maintenance of energy homeostasis. These pathways are also regulated by CaMKK2 and TGF-β-activated kinase-1 (TAK1), which are known to be activated by stress^27^. Xing et al.^7^ reported that AMPK mediates cellular metabolic pathways in skeletal muscles. In the present study, the enriched KEGG pathways were related to apoptosis, stress response, glucagon signaling pathways, fructose and mannose metabolism, protein metabolism, and calcium signaling pathways. Qin et al.^28^ also observed that AMPK, PI3-Akt, MAPK, and FOXO pathways were involved in fat, protein, carbohydrate, and glycolipid metabolism in sheep subjected to nutrition deprivation stress. Studies conducted in magpie birds and duroc pigs have further confirmed that AMPK and MAPK pathways play an important role in maintaining energy homeostasis and physiological stress responses^29,30^.

Several genes and pathways were significantly upregulated in both blood and meat samples in our comparisons. Two such enriched genes in both samples were PTX3 and GADD45. Belonging to the humoral innate immune system, PTX3 is a pattern recognition molecule that is produced by phagocytes and stromal cells at inflammatory sites in response to conditions such as tissue damage and infection^31^. The GADD45 family of genes encode proteins that play crucial roles as stress sensors that regulate and coordinate the response of cells to a number of physiological and environmental stressors resulting in cell cycle arrest, DNA repair, cell survival or apoptosis^32^. Significantly upregulated biological process terms in our treatment comparisons in both blood and muscle samples include PTPN1, HYAL2, SOCS (suppressor of cytokine signaling proteins) and KLF (Kruppel-like factors). The PTPN1 (protein tyrosine phosphatase) is an enzyme linked to endoplasmic reticulum stress response that negatively regulates insulin and leptin and causes insulin and leptin resistance^33^. The membrane-anchored protein, HYAL-2, present in lysosome is essential for hyaluronan metabolism and platelet generation^34^. Numerous KLFs have been identified that are involved in the development and homeostasis of several organ systems in mammals. This family of transcription factors regulates various biological processes, including proliferation, differentiation, growth, development, survival and responses to external stress^35^. In addition, autophagy was among the top 20 upregulated pathways in both blood and muscle samples in our study. Continuous oxidative, mechanical, and heat stresses on skeletal muscle increase protein degradation that necessitates constant protein turnover for healthy state and functioning of muscle^36^. Autophagy is an important pathway by which damaged proteins are separated in vesicles called autophagosomes, which subsequently fuse with lysosomes, where the components are degraded and recycled^37^. Autophagy is also induced by nutritional stress such as starvation and is mediated by Akt/FOXO3 pathway in vivo^38^. Nutrition stress results in FOXO3 phosphorylation because of the absence of insulin and growth factor dependent signaling and inhibition of Akt. FOXO3 then initiates transcription of genes related to autophagy, which is supported by AMPK^39^.

There are conflicting reports regarding appropriate time of muscle sample collection postmortem for best RNA quality in animals. Malila et al.^40^ recommended that muscle samples should be collected within 20 min postmortem, while Bahar et al.^41^ suggested that for RNA gene expression studies, muscle samples can be collected even after weeks of postmortem storage at refrigeration temperature. In our study, CASP7 and FOXO3 genes, both related to apoptosis pathways, were significantly upregulated in muscle samples collected at both 15 min and 6 days postmortem in all comparisons. However, the JAK-STAT signaling pathway was significantly expressed only in muscle samples collected at 6 days postmortem from both 30-min and 180-min transported goats. The Janus kinase-signal transducer and activator of transcription (JAK-STAT) pathway plays a significant role in conveying signals from receptors of cell membranes to the nucleus^42^. This pathway is also involved in regulation of cell differentiation and proliferation, survival, apoptosis, inflammation, and immune response^43^. While highly active transcription occurs in skeletal muscle during the immediate 48 h postmortem period as a result of oxidative stress^44^, Pozhitkov et al.^45^ reported that mRNA transcripts of certain genes became significantly more abundant postmortem in healthy animals, with some transcripts increasing only after 24 and 48 h postmortem. The time of increase and duration of increased abundance of apoptotic gene expressions postmortem may vary in different animals^45^. Sanoudou et al.^44^ suggested that a possible coordinated response may occur in cells based on significant expressions of DNA repair genes such as GADD45A and RAD51 in response to DNA damage and upregulation of apoptosis, proteolysis, and antioxidant activities-related transcripts postmortem.

The release of acute and long-acting stress hormones and their actions on receptors in cells are important factors that initiate the change in gene expression altering the cellular homeostasis in response to stress^46^. After immediate release of epinephrine in response to stress as seen in the current study, the glycogen reserves that are broken down in muscle and liver are restored by glucocorticoids by activating gluconeogenesis. The GO terms related to stress response, inflammatory response, apoptotic response, muscle proteolysis, exosome (RNase complex), kinase activity, and cytokine receptor binding were significantly enriched in our study. Similar DEGs were identified in pigs subjected to cortisol treatment^10^. Cellular response to corticosteroid stimulus (KLF9, MSTN, NR3C1) was also among the significantly upregulated biological processes in GO terms in muscle samples. Wan et al.^10^ observed that cortisol causes significant degree of muscle damage in pigs, and Duclos et al.^47^ found that stress causes severe structural damage to muscle bundles that results in inferior meat quality in poultry. Glucocorticoids are also involved in the apoptosis mechanism^48^ through the activation of caspases^49^.

Our study also revealed that CASP7 expression was significantly upregulated among the DEGs in a majority of comparisons in both blood and muscle samples. The caspase family of genes are involved in signaling pathways of apoptosis, necrosis, and inflammation. Caspase 3, caspase 7 and caspase 9 are involved in the cell death^50^. Increased stress hormone concentrations in animals likely cause increased cell apoptosis, as evidenced by the structural damage to muscle tissue and meat quality deterioration reported by other authors and as supported by our study. Catabolic conditions result in cytokines or insulin resistance and activation of caspases^51^. Upregulation of PTPN1 and SOCS due to stress as noticed in the present study could have been involved in insulin and cytokine resistance and the resultant caspases activity. Furthermore, caspase-3 has been reported to breakdown actomyosin producing actin fragments and other proteins degraded by the ATP-ubiquitin-proteosome system^51^. The authors suggested that activation of caspase-3 could be the initial step in catabolic conditions in live organisms. Protein degradation in rats is uninterrupted under catabolic conditions even when the calcium-activated proteases are blocked, suggesting that calpains are not involved in early breakdown of proteins^52^. Caspase activation has also been identified as the primary mechanism in postmortem muscle proteolysis, and the process of conversion of muscle to meat is suggested to begin with apoptosis, which is interrelated to proteolysis^6^. This interrelationship is evident by the fact that calpain-1 plays an important role in modulating apoptosis by activating caspase 3^53^, which in turn disintegrates the calpain-inhibitor, calpastatin^54^. While severe oxidative stress may also promote apoptosis and subsequent proteolysis resulting in meat tenderization, apoptosis occurring early postmortem may adversely affect organoleptic properties of meat due to its negative association with antioxidant metabolites^55^. Our study supports the earlier suggestion by Goncalves et al.^56^ that the differential gene networks related to proteolysis, calcium signaling, and apoptosis appear to be potential regulators of meat tenderness.

In addition to caspases, other endogenous proteolytic enzymes play important roles in postmortem meat tenderization process, which include calpains, cathepsins and calpastatin^57^, all of which require specific pH ranges for optimal activities. In our study, desmin degradation of goat meat during aging was associated with ultimate pH. Among the endogenous proteolytic enzymes, μ-calpain plays a major role in postmortem proteolysis and tenderization of meat^58^. In an experiment conducted to study the effect of nutritional levels on meat quality, it was found that the expression levels of calpastatin (CAST) and calpain significantly affected the tenderness of meat, indicating that CAST and calpain gene expressions play vital roles in determining meat quality characteristics^59^. In our study, the calcium/calmodulin-dependent protein kinase type II delta (CAMK2D), which is part of a complex that regulates calcium influx in skeletal muscle^60^, as well as the genes related to response to endoplasmic reticulum stress (PARP16, PTPN1, TMTC3, TOR1A, STT3B, DNAJB9, ERLEC1, EIF2S1, UBE2J2, FAF2), were upregulated and this could also be related to the activity of the calpain/calpastatin enzyme system. Desmin levels at 6 days postmortem was positively correlated with calpastatin activity at 24 h postmortem in our study.

The heat shock protein family A (Hsp 70) member 12A (HSPA12A) was also a significantly upregulated DEG in muscle samples in our TRT comparisons. Small HSPs block the release of cytochrome c from mitochondria and prevent activation of caspases in order to maintain homeostasis in muscle fibers^61^. In a study using Nellore cattle, Carvalho et al.^62^ reported that HSP 27 and HSP70 have antitopototic activity; however, HSP27 may increase tenderness and HSP70 may decrease tenderness of meat. Meat tenderness in cattle is influenced by several genes of the HSP family and other genes such as those involved in energy metabolism^63^. HSPB1 has been reported to be negatively correlated with tenderness in beef^63^. The increased WBSF values in the transported groups compared to the control group and the significant enrichment of the HSPA12A gene in muscle samples may indicate an inverse relationship between this family of genes and meat tenderness.

## Conclusions

The effect of transportation stress on meat quality was analyzed phenotypically and using RNA-seq methodology to further understand the intrinsic mechanisms at the molecular level. The primary focus was on the genes and pathways related to stress and meat quality characteristics. Both postmortem muscle calpastatin and cooked meat shear values increased with transportation time. The effect of stress on desmin levels (day 6) was similar to that on calpastatin and WBSF values. The positive correlation between desmin concentration at day 6 and pH, calpastatin, and WBSF at day 1 indicates the complex interaction among ultimate pH, activities of endogenous proteolytic enzymes, desmin degradation, and development of meat tenderness. Biological processes and DEGs related to cellular response to corticosteroid stimulus (ex. KLF9), energy homeostasis (ex. AMPK, FOXO), response to stress and cope up mechanisms (PTX3, GADD45), endoplasmic reticulum stress and insulin resistance (ex. PTPN1) appear to be enriched because of transportation stress. In addition, DEGs, such as CASP7, CFLAR, DDIT4, MAPK4, and HSPA12A that were significantly enriched are related to proteolytic and apoptosis pathways and appear to be an effect of animal stress on meat quality. Biological processes and differential gene network related to endoplasmic reticulum stress and calcium influx that were enriched in our study suggest that calpain/calpastatin system is also involved in determining meat tenderness during aging as supported by the positive correlation between muscle calpastatin and desmin. Some DEGs related to apoptosis appear to be enriched particularly during extended postmortem storage of meat (ex. JAK-STAT). The AMPK pathways that are crucial for maintenance of energy homeostasis are likely involved in both stress response and mediating cellular metabolic pathways in skeletal muscles. Therefore, there is evidence in our study that stress hormones induced apoptosis of skeletal muscles cells by activating capsases and related signaling pathways. Pre-slaughter stress in goats leads to a sequence of events at cellular and molecular levels that is reflected in the quality of meat, particularly in tenderness-related variables.

## Supplementary Information

Supplementary Information.
