# Supplementary material for: RNA-Seq exploration of the influence of stress on meat quality in Spanish goats
Source: Sci Rep. 2022 Nov 29;12:20573. doi: 10.1038/s41598-022-23269-8 (PMC9709060; doi:10.1038/s41598-022-23269-8)
Supplement: Supplementary file 1 — Supplementary Information. [file 41598_2022_23269_MOESM1_ESM.pdf]

Supplementary Information

RNA-Seq Exploration of the influence of Stress on Meat Quality in Spanish Goats

Aditya Naldurtiker, Phaneendra Batchu, Brou Kouakou, Thomas H. Terrill,
Arshad Shaik, Govind Kannan*

Agricultural Research Station, Fort Valley State University, Fort Valley, GA 31030

Corresponding Author: Govind Kannan, Ph.D.
Professor of Animal Science
Agricultural Research Station
Fort Valley State University
1005 State University Drive
Fort Valley, GA 31030
Phone: (478) 825-4613
Fax: (478) 827-3101

TRT	PM Time[4]	FPKM interval				
		**0-1**	**1-3**	**3-15**	**15-60**	**>60**
Control[1]	15 min	16,669 (61.07 %)	2,808 (10.30 %)	4,192 (15.37 %)	2,201 (8.07 %)	1,426 (5.23 %)
Control	6 days	16,453 (60.33 %)	2,971 (10.89 %)	4,410 (16.17 %)	2,152 (7.89 %)	1,286 (4.72 %)
30 min[2]	15 min	16,593 (60.84 %)	2,688 (9.86 %)	4,260 (15.56%)	2,310 (8.47 %)	1,422 (5.20 %)
30 min	6 days	16,440 (60.29 %)	2,886 (10.58 %)	4,446 (16.30 %)	2,191 (8.04 %)	1,307 (4.79 %)
180 min[3]	15 min	16,885 (61.91 %)	2,747 (10.07 %)	4,007 (14.69 %)	2,186 (8.01 %)	1,447 (5.31 %)
180 min	6 days	16,697 (61.23 %)	2,887 (10.59 %)	4,214 (15.45 %)	2,130 (7.86 %)	1,343 (4.92 %)

Supplementary Table T1. Fragments per kilobase of exon per million mapped reads (FPKM) of *Longissimus dorsi* muscle samples from carcasses of goats subjected to transportation stress treatment (TRT). [1]Not transported but held in pens; [2]Transported for 30 min; [3]Transported for 180 min; [4]Postmortem (PM) sample collection time (15 min or after 6 days of aging).

Gene ID	Log2fold change	*P*-value	Gene name	Gene description
Upregulated – 15 min postmortem				
ENSCHIG00000011503	1.116343012	0.0000062	CASP7	Caspase 7
ENSCHIG00000012523	1.562908845	0.00073	PTX3	Pentraxin 3
ENSCHIG00000013864	1.166767915	0.0000071	G3BP2	G3BP stress granule assembly factor 2
ENSCHIG00000009880	0.904655719	0.000154	CAMK2D	Calcium/calmodulin dependent protein kinase II delta
ENSCHIG00000018293	1.289488892	0.0001	FOXO3	Forkhead box O3
Downregulated – 15 min postmortem				
ENSCHIG00000005813	-0.774442042	0.0000064	VSIR	V-set immunoregulatory receptor
ENSCHIG00000018532	-1.018371932	0.00000716	COPRS	Coordinator of PRMT5 and differentiation stimulator
ENSCHIG00000018789	-0.749470369	0.0000135	SLC12A7	Solute carrier family 12-member 7
ENSCHIG00000026577	-0.61898422	0.0000115	RIPK1	Receptor interacting serine/threonine kinase 1
ENSCHIG00000004895	-1.318038956	0.0000108	STARD5	StAR related lipid transfer domain containing 5
Upregulated – 6 days postmortem				
ENSCHIG00000011503	0.902595388	0.000337	CASP7	Caspase 7
ENSCHIG00000018293	1.091474566	0.001758	FOXO3	Forkhead box O3
ENSCHIG00000002992	0.99916149	0.002955	HSPA12A	Heat shock protein family A (Hsp70) member 12A
ENSCHIG00000017239	0.76116458	0.009037	OXSR1	Oxidative stress responsive 1
ENSCHIG00000014436	1.074646224	0.012237	PRKAA2	Protein kinase AMP-activated catalytic subunit alpha 2
Downregulated – 6 days postmortem				
ENSCHIG00000020969	-1.369528452	0.0000833	MDK	Midkine
ENSCHIG00000018463	-1.059518328	0.000144	MYCBPAP	MYCBP associated protein
ENSCHIG00000023495	-1.811270042	0.000172	NTN5	Netrin 5
ENSCHIG00000026258	-1.270234943	0.000185	LXN	Latexin
ENSCHIG00000018311	-1.297168938	0.000186	LTB	Lymphotoxin beta

Supplementary Table T2. Differentially expressed genes (DEGs) in loin/rib chops (*Longissimus dorsi*) sampled at 15 min postmortem or after 6 days of aging from 30 min vs. control comparison. The DEGs shown are closely associated with both stress responses and meat quality.

Gene ID	Log2fold change	*P*-value	Gene name	Gene description
Upregulated – 15 min postmortem				
ENSCHIG00000018191	0.947642064	0.00023	FOXO1	Forkhead box O1
ENSCHIG00000011503	0.999947556	0.00039	CASP7	Caspase 7
ENSCHIG00000015845	1.083902296	0.00102	PFKFB4	6-phosphofructo-2-kinase/fructose-2,6-biphosphatase 4
ENSCHIG00000016459	0.377036413	0.00652	CFLAR	CASP8 and FADD like apoptosis regulator
ENSCHIG00000006945	0.451703957	0.01754	CDK2	Cyclin-dependent kinase 2
Downregulated – 15 min postmortem				
ENSCHIG00000019714	-0.728178036	0.0000012	RASSF4	Ras association domain family member 4
ENSCHIG00000026170	-1.968406239	0.0000015	SCN2B	Sodium voltage-gated channel beta subunit 2
ENSCHIG00000023475	-2.405196366	0.0000023	PSAT1	Phosphoserine aminotransferase 1
ENSCHIG00000023405	-0.988576577	0.0000026	ZNF827	Zinc finger protein 827
ENSCHIG00000023964	-1.925594775	0.0000048	KY	Kyphoscoliosis peptidase
Upregulated – 6 days postmortem				
ENSCHIG00000014378	0.865767037	0.00892	WNT5B	Wnt family member 5B
ENSCHIG00000018191	1.093446139	0.000013	FOXO1	Forkhead box O1
ENSCHIG00000011503	0.703078551	0.00052	CASP7	Caspase 7
ENSCHIG00000016621	1.060819565	0.00448	GADD45G	Growth arrest and DNA damage inducible gamma
ENSCHIG00000027180	0.283415862	0.01308	GSK3A	Glycogen synthase kinase 3 alpha
Downregulated – 6 days postmortem				
ENSCHIG00000023567	-1.981053548	0.000009	CCL2	C-C motif chemokine ligand 2
ENSCHIG00000024070	-1.060083352	0.0000087	VEGFC	Vascular endothelial growth factor C
ENSCHIG00000025189	-1.114171923	0.0000031	FAM110B	Family with sequence similarity 110 member B
ENSCHIG00000020106	-0.953500523	0.000014	DAGLA	Diacylglycerol lipase alpha
ENSCHIG00000025823	-1.864260853	0.00001	ADAMTS13	ADAM metallopeptidase with thrombospondin type 1 motif 13

Supplementary Table T3. Differentially expressed genes (DEGs) in loin/rib chops (*Longissimus dorsi*) sampled at 15 min postmortem or after 6 days of aging from 180 min vs. control comparison. The DEGs shown are closely associated with both stress responses and meat quality.

**Gene ontology**	**Description**	***P*-value**	**Gene name**
Upregulated			
GO:0051603	Proteolysis involved in cellular protein catabolic process	0.000505	BAG5, TP53INP2, hip2, TMTC3, TOR1A, PDCL3, PSME3, STT3B, DNAJB9, CSNK1D
GO:0034976	Response to endoplasmic reticulum stress	0.003785	PARP16, PTPN1, TMTC3, TOR1A, STT3B, DNAJB9, ERLEC1, EIF2S1, UBE2J2, FAF2
GO:0032869	Cellular response to insulin stimulus	0.0058774	PTPN11, PDK4, APPL1, PIK3R1, MSTN, SOCS7, EIF4EBP2, IRS2, PDPK1, PIK3R3
GO:2001233	Regulation of apoptotic signaling pathway	0.036212	BAG5, HMOX1, HYAL2, PTPN1, SIAH2, PSME3, BNIP3, GSK3A, CYLD, RRN3, HERPUD1
GO:0071384	Cellular response to corticosteroid stimulus	0.0419569	KLF9, MSTN, NR3C1
Downregulated			
GO:0050776	Regulation of immune response	0.000894	NR1D1, CD40, IL15, KIT, NLRC5, FGR, ADAM8, CADM1, IDO1, SMAD3, PIK3R6
GO:0001817	Regulation of cytokine production	0.000754	CD40, IL15, FGR, ADAM8, CADM1, IDO1, SMAD3, NOS2, ARRB2, ISG17, BPI
GO:0070663	Regulation of leukocyte proliferation	0.0010992	IL7, PTPN6, CD74, AIF1, CD40, LMO1, IRF1, PRDM1, CORO1A, CARD11
GO:0030217	T cell differentiation	0.0014473	VAV1, IL7, CD74, KIT, CD4, IRF1, FCER1G, PRDM1, SOX4, PIK3R6, CARD11
GO:0051494	Negative regulation of cytoskeleton organization	0.001896	KANK1, PHLDB2, SCIN, ARHGAP6, STMN2, DLC1, NAV3, ARHGEF2, NEK2, GMFG

Supplementary Table T4. Upregulated and downregulated biological processes in gene ontology (GO) terms of loin/rib chops (*Longissimus dorsi*) from goats subjected transportation stress vs. goats not transported (control).

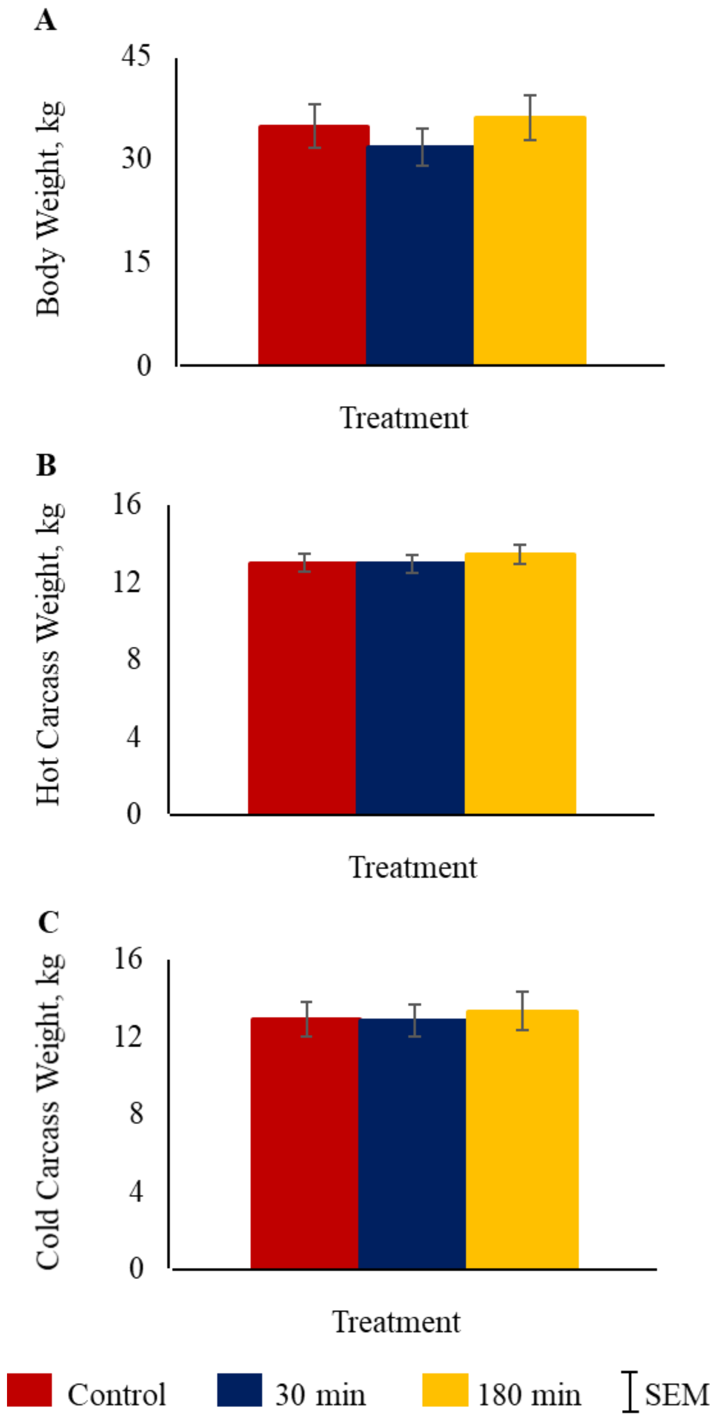

Supplementary Figure S1. Effects of stress treatment (Control = not transported but held in pens; 30 min = transported for 30 min; 180 min = transported for 180 min) on mean ± SEM (A) live, (B) hot carcass, and (C) cold carcass weights in goats.

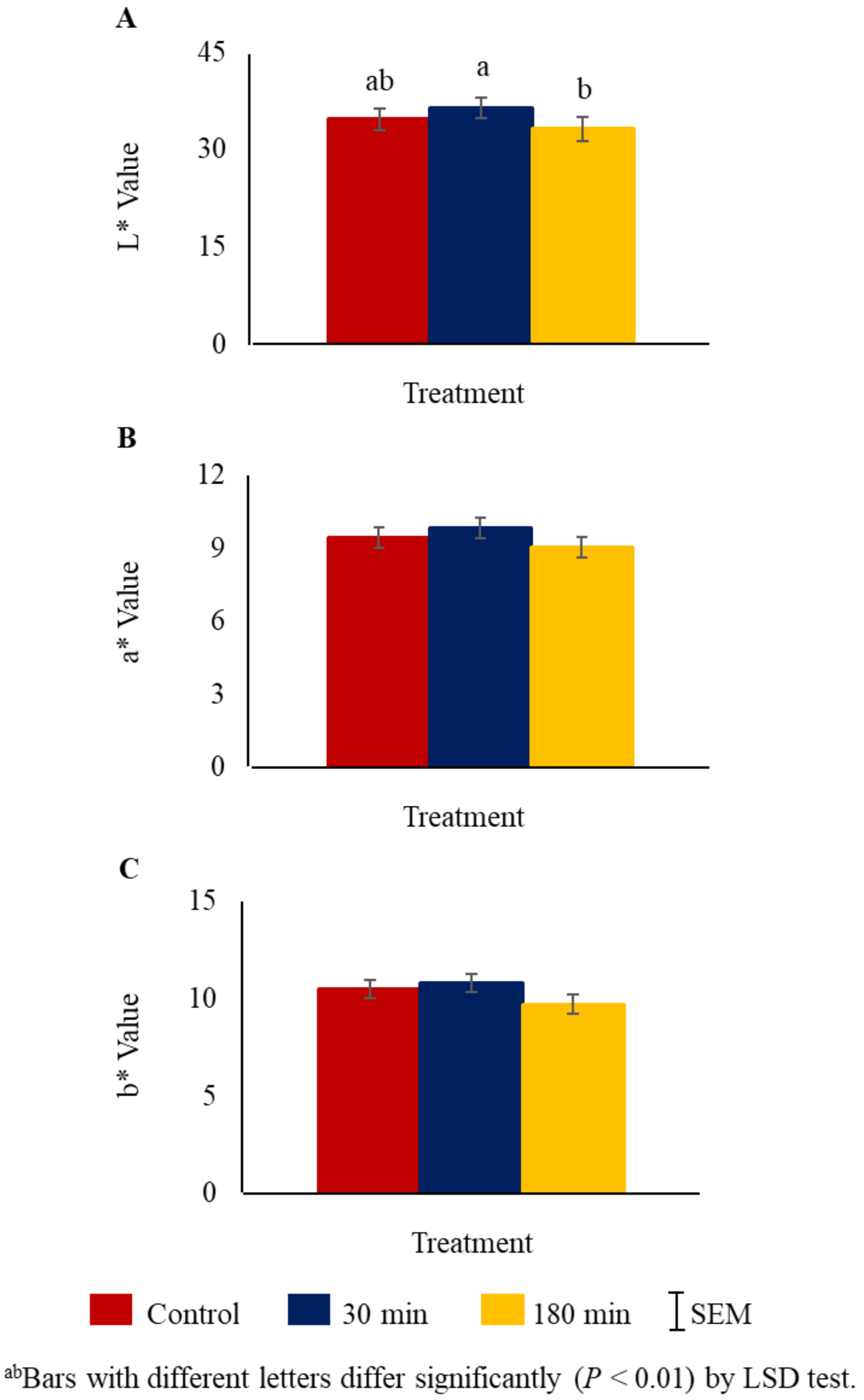

Supplementary Figure S2. Effects of stress treatment (Control = not transported but held in pens; 30 min = transported for 30 min; 180 min = transported for 180 min) on mean ± SEM loin/rib (*Longissimus dorsi)* chop (A) L* (B) a*, and (C) b* values determined at 24 h postmortem in goats.

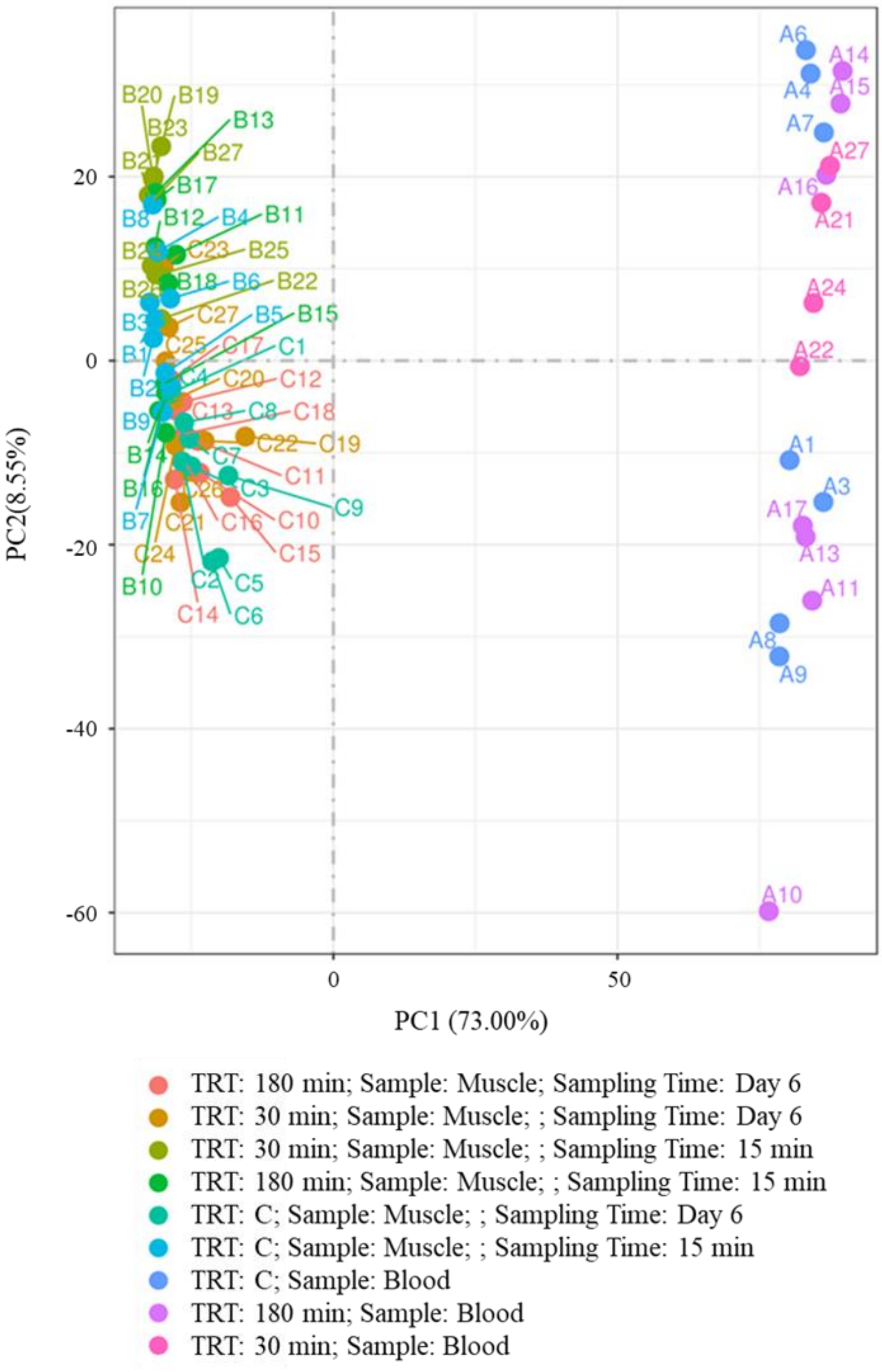

Supplementary Figure S3. Principal component analysis (PCA) of blood samples from goats subjected to stress treatments (TRT: control, C = not transported but held in pens; 30 min = transported for 30 min; or 180 min = transported for 180 min) and of meat samples (*Longissimus dorsi*) at 15 min postmortem or after 6 days.

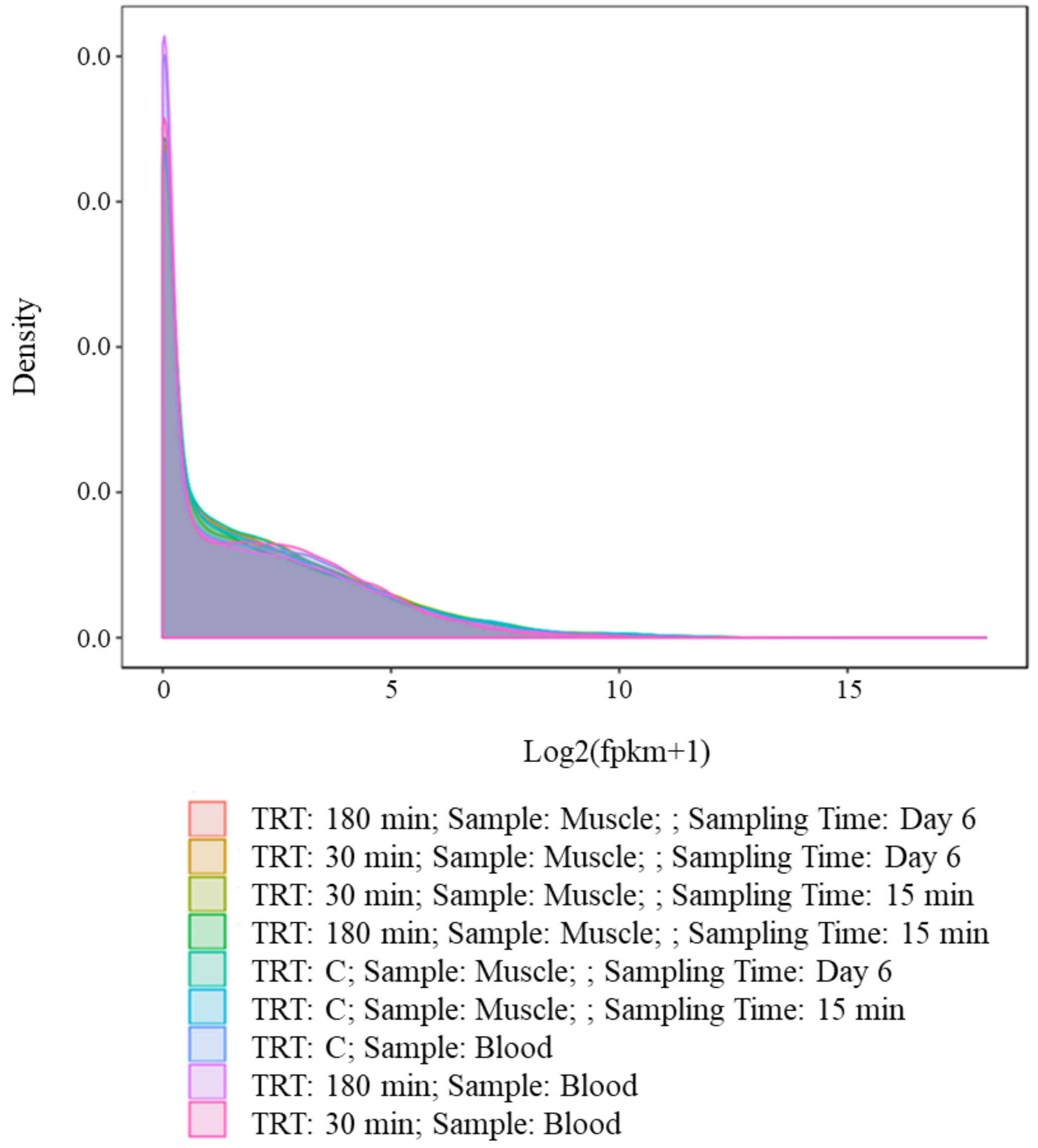

Supplementary Figure S4. Fragments per kilobase of exon per million mapped fragments (FPKM) density distribution of blood samples from goats subjected to stress treatments (TRT: control, C = not transported but held in pens; 30 min = transported for 30 min; or 180 min = transported for 180 min) and of meat samples (*Longissimus dorsi*) collected at 15 min postmortem or after 6 days.

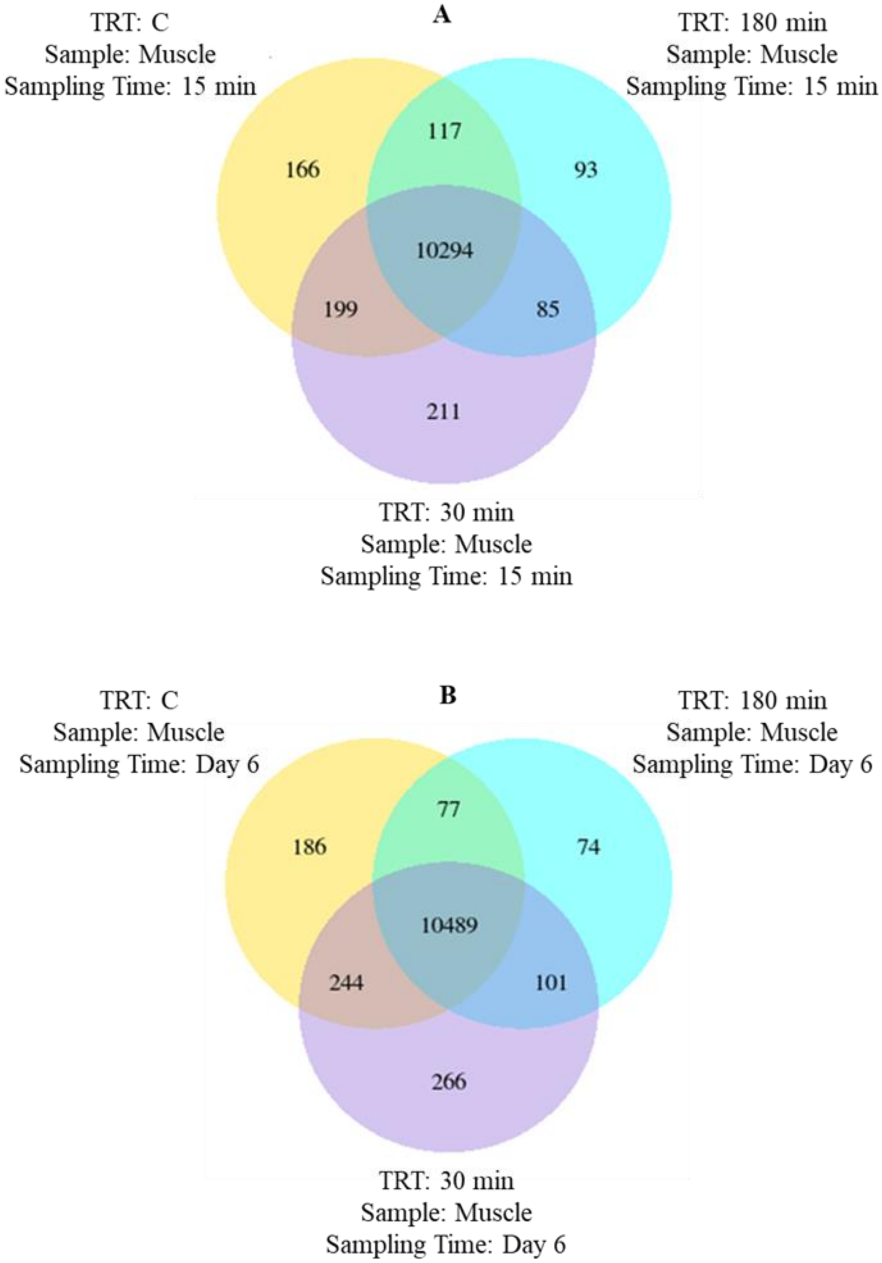

Supplementary Figure S5. Venn diagram showing the co-expression of genes in meat samples (*Longissimus dorsi*) aged for (A) 1 day or (B) 6 days from goats subjected to stress treatments (TRT: control, C = not transported but held in pens; 30 min = transported for 30 min; or 180 min = transported for 180 min).

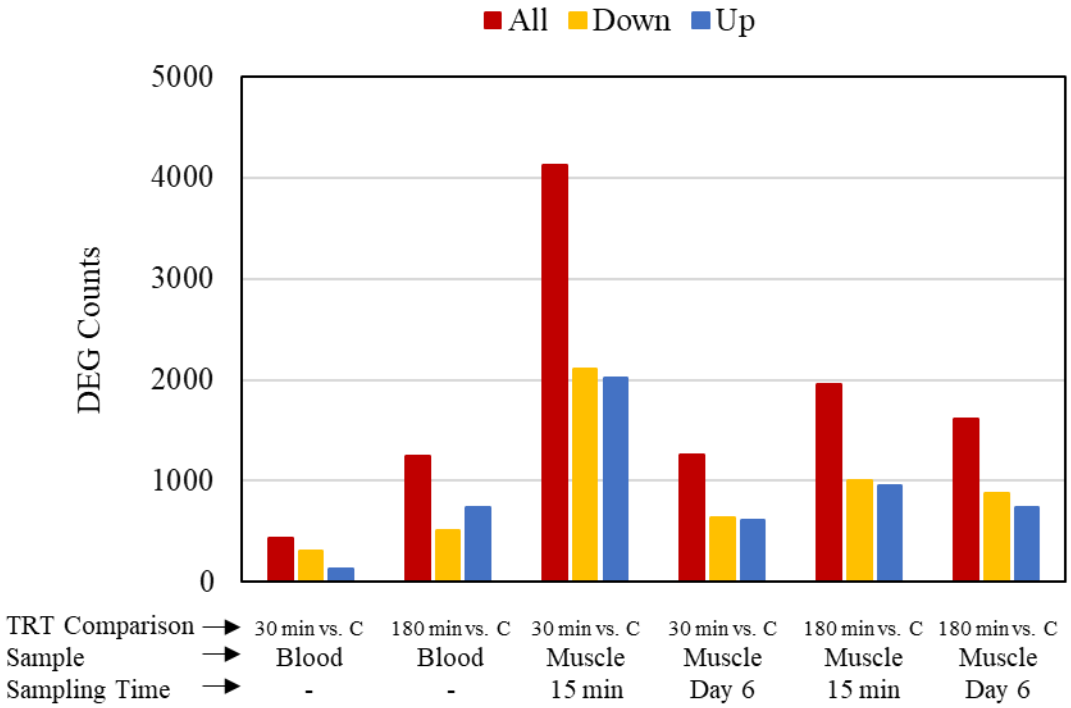

Supplementary Figure S6. Differentially expressed genes of blood samples from goats subjected to stress treatments (TRT: control, C = not transported but held in pens; 30 min = transported for 30 min; or 180 min = transported for 180 min) and of meat samples (*Longissimus dorsi*) at 15 min postmortem or after 6 days.

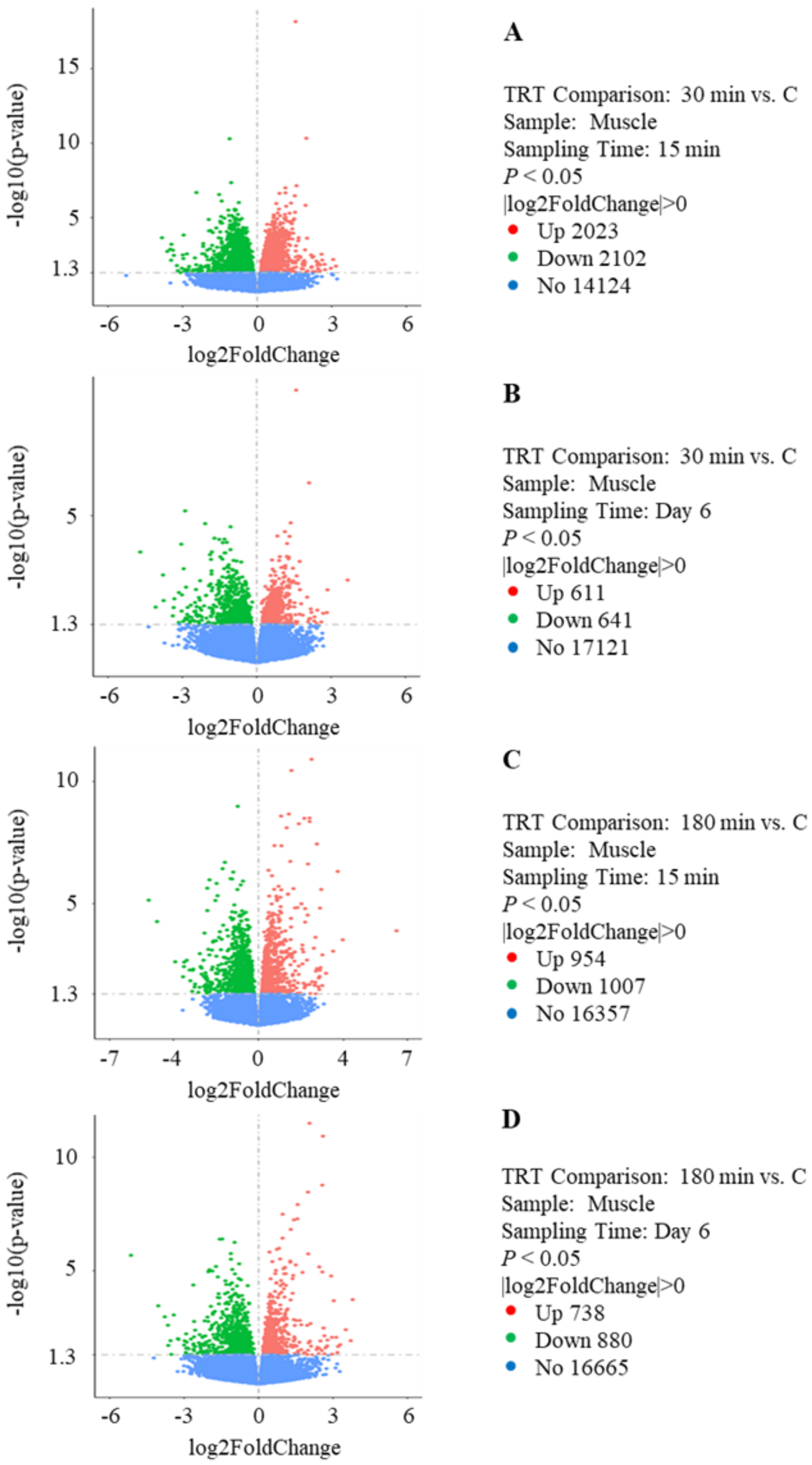

Supplementary Figure S7. Volcano plot of differentially expressed genes (DEG) of meat (*Longissimus dorsi*) samples collected at (A) 15 min postmortem from goats subjected to control (C, not transported but held in pens) vs. 30 min (transported for 30 min), (B) 6 days from control vs. 30 min, (C) 15 min postmortem from control vs. 180 min (transported for 180 min), and (D) 6 days from control vs. 180 min treatments (TRT).

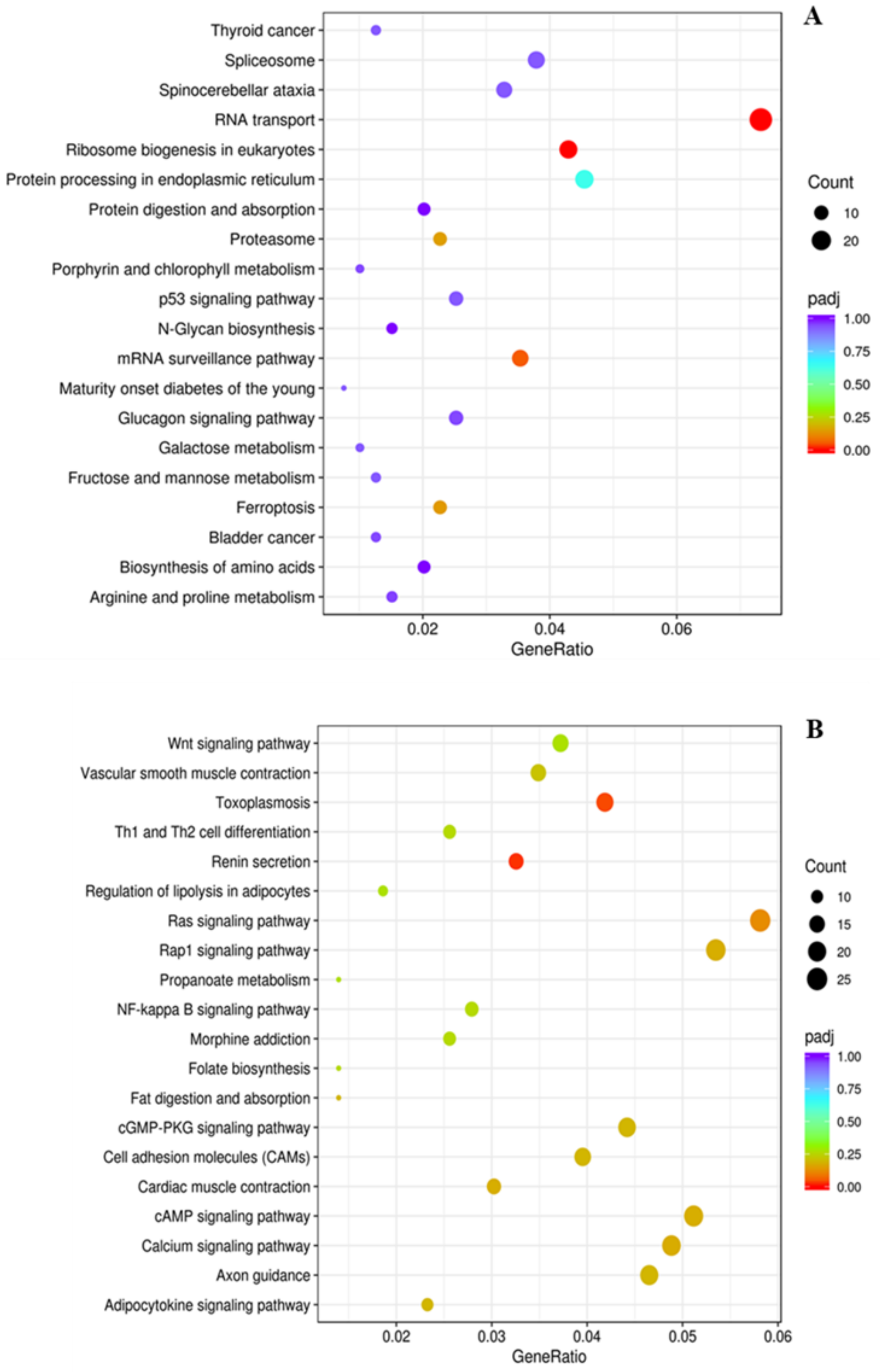

Supplementary Figure S8. (A) Upregulated and (B) downregulated KEGG pathways in meat (*Longissimus dorsi*) samples collected at 15 min postmortem from goats subjected to control (not transported but held in pens) versus 180 min (transported for 180 min) stress treatments.

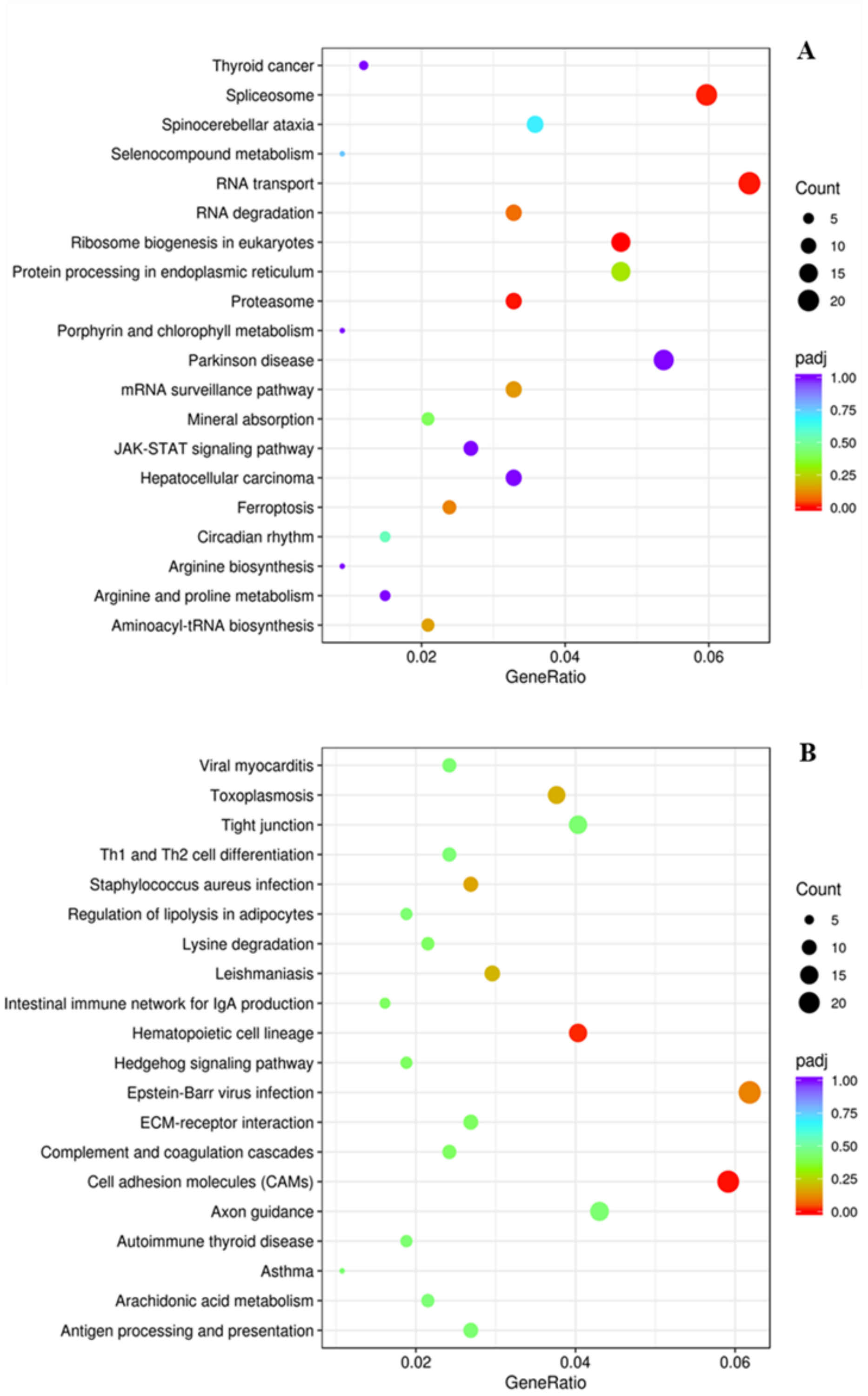

Supplementary Figure S9. (A) Upregulated and (B) downregulated KEGG pathways in meat (*Longissimus dorsi*) samples collected at 6 days postmortem from goats subjected to control (not transported but held in pens) versus 180 min (transported for 180 min) stress treatments.
